# Examining Neurosteroid-Analogue Therapy in the Preterm Neonate For Promoting Hippocampal Neurodevelopment

**DOI:** 10.3389/fphys.2022.871265

**Published:** 2022-04-19

**Authors:** Julia C. Shaw, Rebecca M. Dyson, Hannah K. Palliser, Ryan P. Sixtus, Heather Barnes, Carlton L. Pavy, Gabrielle K. Crombie, Mary J. Berry, Jonathan J. Hirst

**Affiliations:** ^1^ School of Biomedical Sciences and Pharmacy, University of Newcastle, Newcastle, NSW, Australia; ^2^ Mothers and Babies Research Centre, Hunter Medical Research Institute, Newcastle, NSW, Australia; ^3^ Department of Paediatrics and Child Health, University of Otago, Wellington, New Zealand; ^4^ Centre for Translational Physiology, University of Otago, Wellington, New Zealand

**Keywords:** neurosteroids, preterm neonatal brain, oligodendrocyte, GABA, glutamate, neuron

## Abstract

**Background:** Preterm birth can lead to brain injury and currently there are no targeted therapies to promote postnatal brain development and protect these vulnerable neonates. We have previously shown that the neurosteroid-analogue ganaxolone promotes white matter development and improves behavioural outcomes in male juvenile guinea pigs born preterm. Adverse side effects in this previous study necessitated this current follow-up dosing study, where a focus was placed upon physical wellbeing during the treatment administration and markers of neurodevelopment at the completion of the treatment period.

**Methods:** Time-mated guinea pigs delivered preterm (d62) by induction of labour or spontaneously at term (d69). Preterm pups were randomized to receive no treatment (Prem-CON) or ganaxolone at one of three doses [0.5 mg/kg ganaxolone (low dose; LOW-GNX), 1.0 mg/kg ganaxolone (mid dose; MID-GNX), or 2.5 mg/kg ganaxolone (high dose; HIGH-GNX) in vehicle (45% β-cyclodextrin)] daily until term equivalence age. Physical parameters including weight gain, ponderal index, supplemental feeding, and wellbeing (a score based on respiration, activity, and posture) were recorded throughout the preterm period. At term equivalence, brain tissue was collected, and analysis of hippocampal neurodevelopment was undertaken by immunohistochemistry and RT-PCR.

**Results:** Low and mid dose ganaxolone had some impacts on early weight gain, supplemental feeding, and wellbeing, whereas high dose ganaxolone significantly affected all physical parameters for multiple days during the postnatal period when compared to the preterm control neonates. Deficits in the preterm hippocampus were identified using neurodevelopmental markers including mRNA expression of oligodendrocyte lineage cells (*CSPG4*, *MBP*), neuronal growth (*INA*, *VEGFA*), and the GABAergic/glutamatergic system (*SLC32A1*, *SLC1A2*, *GRIN1*, *GRIN2C*, *DLG4*). These deficits were not affected by ganaxolone at the doses used at the equivalent of normal term.

**Conclusion:** This is the first study to investigate the effects of a range of doses of ganaxolone to improve preterm brain development. We found that of the three doses, only the highest dose of ganaxolone (2.5 mg/kg) impaired key indicators of physical health and wellbeing over extended periods of time. Whilst it may be too early to see improvements in markers of neurodevelopment, further long-term study utilising the lower doses are warranted to assess functional outcomes at ages when preterm birth associated behavioural disorders are observed.

## Introduction

Despite significant efforts, preterm birth in Australia and other resource-rich nations continues to impact around 8–10% of newborns, with even higher rates reported in resource-limited settings. With increased survival into childhood and adult life, the adverse impact of preterm-associated brain injury has become increasingly clear yet there are no targeted postnatal therapies to either mitigate or prevent its occurrence (Beck et al., 2010; Singh et al., 2013; Morris et al., 2020). Compared to their term-born peers, children born preterm are at an increased risk of developing behavioural disorders such as anxiety and attention deficit hyperactivity disorder (ADHD), in addition to reduced academic attainment at school (Chyi et al., 2008; Lindström et al., 2011; Peralta-Carcelen et al., 2018). It is well established that deficits in white matter microstructure are a leading contributor to the development of these neurodevelopmental disorders, with evidence emerging to suggest that an imbalance between the inhibitory GABAergic and excitatory glutamatergic pathways plays a key role (Caldinelli et al., 2017; Vollmer et al., 2017; Shaw et al., 2020). Importantly, an integral trophic mechanism of *in utero* neurodevelopment is the GABAergic neurosteroid, allopregnanolone (3α-hydroxy, 5α-pregnane-20-one) (Hirst et al., 2014). Allopregnanolone is synthesised from placentally-derived progesterone, either within the placenta itself, or within the fetal brain. As such, it is found in very high concentrations in the fetal plasma and brain compared to concentrations in the neonate (Bičíková et al., 2002; Kelleher et al., 2013). By acting on GABA_A_ receptors throughout the fetal brain, allopregnanolone promotes the fetal ‘sleep-like’ state by increasing GABAergic inhibitory tone (Nicol et al., 2001). Furthermore, allopregnanolone also promotes the maturation of immature oligodendrocytes and the production of myelin by mature oligodendrocytes (Ghoumari et al., 2003; Shaw et al., 2021).

Preterm birth leads to a loss of placentally derived progesterone. As progesterone and other neurosteroids are not exogenously provided to the preterm neonate, the preterm brain must continue on its trajectory of development and maturation without the presence of trophic factors that are key for optimal brain development in a fetus of equivalent post conceptional age. Thus, we propose that following preterm birth, postnatal restoration of allopregnanolone warrants investigation as a novel strategy to protect and optimise neonatal brain development in this high-risk group. However, the administration of allopregnanolone itself comes with some difficulty due to its very short half-life (∼2 h in adult humans), and its potential to be metabolised into other steroids such as its immediate precursor (5α-dihydroprogesterone) which has very low affinity for the GABA_A_ receptor, or isopregnanolone which is a potent GABA_A_ receptor antagonist (Mellon and Griffin, 2002; Timby et al., 2006; Bengtsson et al., 2015). Ganaxolone, as a synthetic, longer-acting analogue of allopregnanolone, may enable the neuroprotective actions of allopregnanolone to be achieved without the complication of steroid metabolism side-effects.

Ganaxolone is a β-methylated analogue of allopregnanolone with similar binding affinity for GABA_A_ receptors as allopregnanolone. It has the advantages of a methyl group which prevents metabolism into other active steroids and a much longer half-life of ∼12–20 h in adult humans (Carter et al., 1997). Whilst still under clinical testing, ganaxolone has shown great promise in neonates as a treatment for various epilepsy disorders and seizures due to its anti-seizure/anti-convulsant properties through its action on extrasynaptic GABA_A_ receptors (Yawno et al., 2017; Bialer et al., 2020; Lattanzi et al., 2021). In line with these observations, preclinical rodent studies have focused on seizure models such as those induced by bicuculline and picrotoxin, where administration of ganaxolone reduces seizure length and severity (Yum et al., 2014; Bialer et al., 2020). Additionally, emergent data from rodent studies highlight how ganaxolone can reduce anxious, aggressive and, or depressive behaviours in neuropathological states such as the socially isolated mouse model of post-traumatic stress disorder (Pinna and Rasmusson, 2014).

Following extensive *in vivo* research using ganaxolone for other applications, the first *in vivo* assessment of its potential to reduce preterm-associated brain injury was undertaken by our group. Between birth and their term equivalent age (‘due date’; TEA) we administered a dose of 5 mg/kg/day to male guinea pigs born moderately preterm (d62 of a 69-days gestation; analogous to a 28–30 weeks gestational age human) (Berry et al., 2015; Shaw et al., 2019). This dose of ganaxolone was intended to mimic *in utero* exposure to allopregnanolone and allow preterm brain development to continue along the same trajectory as it would *in utero*. At 25 days corrected postnatal age (CPNA 25: 25 days after term ‘due date’ and equivalent to early childhood in humans) guinea pigs born preterm or at term underwent a suite of behavioural tests followed by evaluation of white matter development. Importantly, the hyperactive behavioural phenotype observed in the preterm control pups was diminished in the preterm pups that received neonatal ganaxolone therapy, shifting them towards a term phenotype. Furthermore, myelination deficits related to preterm birth in the CA1 region of the hippocampus and overlying subcortical white matter were prevented in the group of animals born preterm but treated with ganaxolone. These results highlight the potential benefits of short-term restoration of GABAergic action in the preterm brain such that behavioural state in later life is more aligned to that of a term-born animal. Crucially, treatment was intended to mitigate the ‘preterm deficit’ (difference in neurosteroid availability between a postnatal preterm individual and a fetus of the same post-conceptional age) and all therapy ceased once TEA was reached. However, the dose used was derived from that used to treat neonatal seizures in a sheep model (Yawno et al., 2017), which resulted in side effects including sedation and respiratory depression during the treatment period. Therefore, in the current study we have used three descending doses; 2.5, 1, and 0.5 mg/kg/day to maximise therapeutic gain free of adverse dose-related side effects. As in the previous study the treatment ended at TEA, at which time the current study was completed to allow for tissue analysis immediately followed the treatment period. Additionally, we also performed this dosing study in female neonates to ensure any sex dependent responses to the treatment were elucidated. Our aim was to identify the ganaxolone treatment approach that would protect the normal developmental trajectory of cerebral myelination such that by TEA minimal differences would be found between preterm and term-born pups.

## Methods

Unless specified otherwise, all reagents were supplied by Sigma Aldrich (Castle Hill, Australia).

### Animals

Mature breeding Dunkin Hartley female guinea pigs were obtained from the University of Otago Wellington Biomedical Research Unit. Guinea pigs were housed indoors under a 12 h light/dark cycle and supplied with standard guinea pig pellets, Specialty Feeds (Glen Forrest, Australia), hay, fresh vegetables, and drinking water supplemented with Vitamin C. Time mated pregnant sows were randomly allocated to either preterm (*n* = 18) or term (*n* = 10) delivery. Term delivery sows received no further intervention, with pups (Term; *n* = 15) delivered spontaneously and receiving no additional support.

Preterm induction of labour was performed as previously described (Berry et al., 2015; Shaw et al., 2016; Shaw et al., 2019). In brief, sows received betamethasone 1 mg/kg subcutaneously (Celestone Chronodose; Merck, Sharp and Dohme, Auckland, New Zealand) 48 and 24 h prior to preterm delivery to accelerate fetal lung maturation and surfactant production. Aglepristone 10 mg/kg (Provet, Auckland, New Zealand) was administered subcutaneously 24 h prior to, and on the morning of delivery to inhibit progesterone-based continuance of pregnancy. Intramuscular oxytocin 3IU/kg (Provet) was administered to stimulate uterine contractions 1 h after the second Aglepristone dose and repeated until all pups and placentas were delivered.

Pups were delivered on d62 of 69-days pregnancy (*n* = 61; see Supp Figure 1 for survival) (Berry et al., 2015) and randomly allocated to standard care only (PREM-CON), vehicle only (PREM-VEH, 45% β-cyclodextrin), or one of three treatment groups. Treated preterm-born pups received either 0.5 mg/kg ganaxolone (low dose; LOW-GNX), 1.0 mg/kg ganaxolone (mid dose; MID-GNX), or 2.5 mg/kg ganaxolone (high dose; HIGH-GNX) in vehicle (45% β-cyclodextrin) split over 2 doses, 12 h apart per day by subcutaneous injection until TEA. No more than 2 pups/sex/sow were used in any allocation. There was no significant difference between the physical parameters for preterm neonates in the PREM-CON and PREM-VEH groups (see Supplementary Figure S2), therefore the PREM-VEH group was not included for further analyses.

**FIGURE 1:**
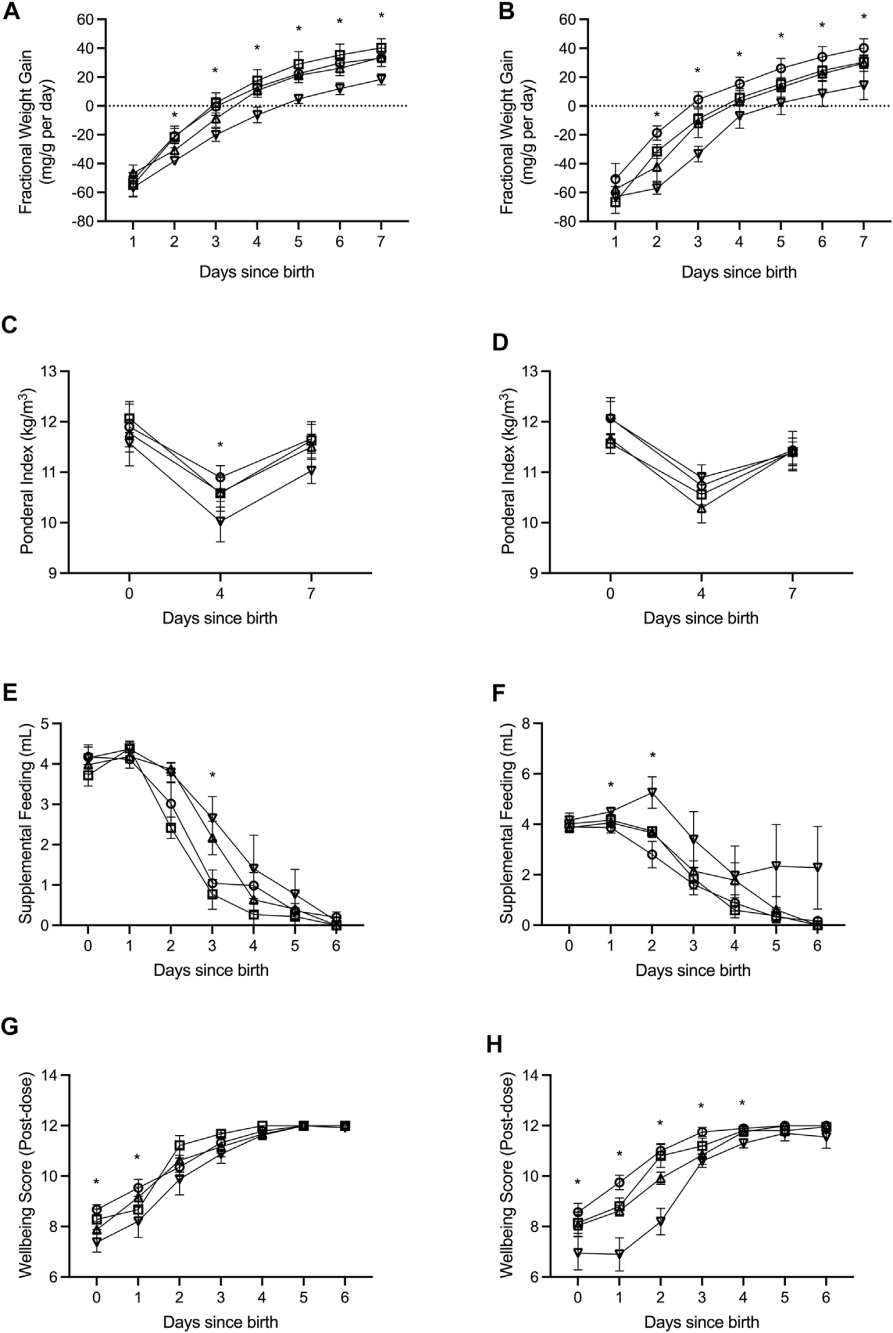
Fractional weight gain **(A,B)**, ponderal index **(C,D)**, supplemental feeding **(E,F)**, and wellbeing score **(G,H)** was recorded from birth (days since birth 0) until term equivalence (postnatal age 7) for male **(A,C,E,G)** and female **(B,D,F,H)** preterm neonates receiving high-GNX (downward facing triangle; males *n* = 6, females *n* = 5), mid-GNX (upward facing triangle; males *n* = 6, females *n* = 6), and low-GNX (square; males *n* = 6, females *n* = 6) and compared to control (circle; males *n* = 7, females *n* = 7) preterm neonates. Data presented as means ± SEM with * indicating significance at *p* < 0.05.

Resuscitation and respiratory support of preterm pups occurred as previously described (Berry et al., 2015; Shaw et al., 2016; Shaw et al., 2019). Respiratory support was provided for at least 3 min to all preterm pups by continuous positive airway pressure (CPAP) support at 5 cm H_2_O using the “Neopuff” t-piece infant resuscitator (Fisher & Paykel, Auckland, New Zealand) with blended oxygen delivered at 5 L/min. All preterm pups were also given an initial fractional inspired oxygen concentration of 30% that was titrated to effect. Once stable respiration was maintained, pups were housed with their mothers and littermates in a warm humidified human infant incubator (Dräger 8000 IC; Drägerwerk AG & Co., Lübeck, Germany); ambient temperature 33°C (titrated down to 28°C by 24 h), and 60% humidity (titrated down to 35% by 12 h).

Preterm pups received 0.2–0.4 ml of Impact guinea pig colostrum replacement (Wombaroo Food Products, Adelaide, Australia) orally within the first hour after birth and then every 2 h until 24 h old. Between postnatal days 1–7 the pups were fed 0.5–1.0 ml of Impact guinea pig milk replacement (Wombaroo Food Products) every 2 h or as needed to supplement independent suckling. Blood sugar levels were measured by ear prick 1 h post drug treatment, and standardised assessments of ‘wellbeing’ (see Table 1) scored 2 h post each drug treatment.

**TABLE 1:** Wellbeing scoring.

Score	Respiration	Posture	Movement
1	Gasping only	Lying down; severe spasticity of neck and front paws	Only when stimulated
2	Gasping with some normal breathing	Can sit up, but difficulty holding head up; some spasticity	Some spontaneous movement
3	No gasping but irregular or shallow breaths	Upright, sitting, can walk but wobbly and uncoordinated	Some activity
4	Normal, fast, and regular breaths	No spasticity, can stand and walk easily	Very active and alert

Scoring for each category was obtained 2 h following drug administration for each neonate each day until term equivalence. Each category was assigned a score from 1–4 (with 1 being the poorest, and 4 being the optimal score). Scores were then added together to give a total out of 12 for each time point.

#### Tissue Collection

Preterm neonates were euthanized at term equivalence, and term neonates within 24 h of birth, by exsanguination under isoflurane, and organ weights recorded. Each brain was sectioned in the sagittal plane to separate the hemispheres. Each left hemisphere was fixed in 4% paraformaldehyde, whilst the right hemisphere was further dissected and frozen in liquid nitrogen.

### Immunohistochemistry

Mature myelinating oligodendrocyte and neuronal nuclei expression was quantified in the CA1 region of the dorsal hippocampus and overlying subcortical white matter as previously described (Shaw et al., 2016; Shaw et al., 2019) by immunohistochemistry performed on 8 μm sections of paraffin-embedded brains cut using a Leica RM2145 Microtome (Leica Microsystems Pty Ltd, North Ryde, Australia). Briefly, dewaxing and rehydrating of tissue sections were performed by xylene and ethanol incubations, antigen retrieval was performed by incubation in citrate buffer (pH 6.0) at 90–95°C for 25 min and cooling for a further 15 min. Blocking for endogenous peroxidases occurred in phosphate buffered saline (PBS) containing 3% hydrogen peroxide for 20 min, and for non-specific staining in a goat serum block (2% goat serum, 0.4% BSA, 0.3% TritonX in PBS) for 1 h. Incubation in primary antibodies at 1:1,000 dilution [myelin basic protein (MBP) M9434; neuronal nuclei (NeuN) MAB377 Millipore] was performed overnight, and incubation in secondary antibodies at 1:300 dilution (biotinylated anti-rat IgG B7139; biotinylated anti-mouse IgG B6649) for 1 h before tertiary incubation in streptavidin-biotin-horseradish peroxidase complex (ab7403 Abcam) at 1:400 dilution for 1 h. All incubations took place at room temperature unless specified. Incubation in 3,3’-diaminobenzidine tetrahydrochloride solution (Metal Enhanced DAB Substrate Kit; ThermoFisher Scientific, Scoresby, Australia) revealed immunolabelling.

Stained slides were digitally imaged using the Aperio imaging system (Leica Biosystems, North Ryde, Australia). ImageJ v1.51 (National Institutes of Health, Bethesda, Maryland) was used to calculate percent area coverage by conversion to grayscale and then binary, and manually adjusting threshold based on the original stained image. Overall average of staining was calculated by taking the average of four images captured from two consecutive sections per animal, i.e., a total of eight images/region/animal.

### Real Time PCR

Frozen hippocampal tissue was prepared for PCR as previously described (Shaw et al., 2016; Shaw et al., 2019; Crombie et al., 2021). Briefly, tissue was homogenised in RLT Plus Buffer (Qiagen RNeasy Plus Mini Kit, Qiagen Pty Ltd, Chadstone, Australia) using a Precellys 24 dual-tissue homogenizer (Bertin Technologies, France). RNA extraction was then performed using the Qiagen RNeasy Plus Mini Kit (Qiagen) by following the manufacturers instructions. Synthesis of cDNA was then performed using the Superscript III Reverse Transcripton Kit (Invitrogen) on a GeneAmp 9700 PCR machine (Applied Biosystems, Life Technologies Pty Ltd, Mulgrave, Australia). cDNA samples were then preamplified on the QuantStudio 6 Flex RT-PCR system (Applied Biosystems) using PreAmp Master Mix (Fluidigm, San Francisco, United States), according to manufacturer’s instructions. Relative mRNA expression analysis was conducted simultaneously on an integrated fluidic chip (Fluidigm). The primer master mix for each primer (0.5 pmol/μl, Fluidigm) and EVAGreen (Bio-Rad Laboratories, Hercules, United States) was used to detect PCR products of the genes of interest (Table 2). RT-PCR was then performed using the Biomark HD system (Fluidigm) and results analysed by RT-PCR analysis software v4.5.2 (Fluidigm) using the comparative CT method of analysis normalised to four housekeeping genes (*ACTB*, *TBP*, *YWHAZ*, *UBE2D2*).

**TABLE 2:** Guinea pig specific primers for real time PCR.

Gene ID	Protein	Forward primer	Reverse primer	Amplicon size (Bp)
*ABAT*	GABA aminotransferase (GABA-T)	GGA​TGT​CCC​AGC​TTG​TCA​CTA	TTG​CTC​CGG​TAC​CAC​ATG​AA	85
*ACTB*	Beta Actin (housekeeper)	TGC​GTT​ACA​CCC​TTT​CTT​GAC​A	ACA​AAG​CCA​TGC​CAA​TCT​CAT	72
*CALB1*	Calbindin	CTG​ACT​GAG​ATG​GCC​AGG​TTA	CCC​ACA​CAT​TTT​AAC​TCC​CTG​AAA	75
*CSPG4*	Chondroitin Sulfate Proteoglycan 4 (NG2)	CTC​CTC​ACC​ACC​ACC​CTC​AA	ACT​CTT​CAG​CAC​AGC​CCT​CA	79
*DLG4*	Postsynaptic density protein 95 (PSD-95)	TAT​TCC​CAG​CAC​CTG​GAC​AA	TCA​TGG​CTG​TGG​GGT​AAT​CA	70
*GABRA1*	GABAA Receptor Subunit Alpha 1	CTC​AAG​CCC​GCA​ATG​AAG​AAA	TCC​AGT​CAA​CGT​GCT​CAG​AA	81
*GABRA2*	GABAA Receptor Subunit Alpha 2	ACT​AGG​CCA​ATC​AAT​TGG​GAA	TCA​AGT​GGA​AAT​GAG​CTG​TCA	80
*GABRA3*	GABAA Receptor Subunit Alpha 3	TTG​GCA​GCT​ATG​CCT​ACA​CA	ACC​TCC​ACA​GAC​TTG​TTC​TTC​C	73
*GABRA4*	GABAA Receptor Subunit Alpha 4	TGG​ACA​AAG​GGT​CCT​GAG​AAA	CAC​TGT​TTG​CCC​AAT​CAG​ATC​A	84
*GABRA5*	GABAA Receptor Subunit Alpha 5	TGG​TTC​ATC​GCT​GTG​TGC​TA	CCC​AGC​CTC​TCT​TCG​TGA​AAT​A	85
*GABRD*	GABAA Receptor Subunit Delta	ATG​CTG​GAC​CTG​GAA​AGC​TA	GGA​TCT​GCT​CCT​GGT​TCT​CA	76
*GABRG2*	GABAA Receptor Subunit Gamma 2	AGG​CAG​ATG​CCC​ATT​GGA​TA	TGT​AGA​GCA​CTC​TGC​CAT​CA	72
*GAD1 (67)*	Glutamate Decarboxylase 1 (67 kDa)	AGC​TCG​CTA​CAA​GTA​CTT​CCC	TGT​GTT​CTG​AGG​TGA​AGA​GGA​C	83
*GAD2*	Glutamate Decarboxylase 2	GGC​GCC​ATC​TCC​AAC​ATG​TA	TGC​CCT​TCT​CCT​TGA​CCT​CA	73
*GLS1*	Glutaminase	CAC​GTT​GGT​CTT​CCT​GCA​AA	GCA​CAT​CAT​GCC​CAT​GAC​A	78
*GRIN1*	Glutamate Ionotropic Receptor 1 (GluN1)	AGA​GCA​TCC​ACT​TGA​GCT​TCC	TAC​ACG​CGC​ATC​ATC​TCG​AA	82
*GRIN2C*	Glutamate Ionotropic Receptor 2C (GluN2C)	ATG​CAC​ACC​CAC​ATG​GTC​AA	CGT​CCA​GCT​TCC​CCA​TCT​TAA	79
*INA*	Alpha-internexin	ACA​AGA​TCA​TCC​GCA​CCA​AC	GTG​CAC​CTT​TTC​GAT​GAA​CAC	80
*MBP*	Myelin Basic Protein	ACC​TCC​TCC​GTC​TCA​AGG​AAA	GCT​CTG​CCT​CCA​TAG​CCA​AA	66
*OLIG2*	Oligodendrocyte Transcription Factor 2	GCA​CTC​ATC​CTG​GGG​ACA​A	CCG​ACG​ACG​TGG​ATG​ATG​AA	78
*PVALB*	Parvalbumin	AAGGATGGGGACGGCAAA	GGG​TCC​ATC​AGC​TCT​GCT​TA	77
*RBFOX3*	RNA Binding Fox-1 Homolog 3 (NeuN)	CAC​AGA​CAG​ACA​GCC​AAC​CA	CGG​AAG​GGG​ATG​TTG​GAG​AC	88
*SLC1A2*	Excitatory amino acid transporter 3 (EAAT3)	CAC​AGT​CGT​CTC​CCT​GTT​GAA	CAG​GCC​CTT​CTT​GAG​AAC​CA	76
*SLC32A1*	Vesicular inhibitory amino acid transporter (vGAT)	ACA​CGA​CAA​GCC​CAA​GAT​CA	TAG​CAC​GAA​CAT​GCC​CTG​AA	76
*SST*	Somatostatin	AAG​CAG​GAA​CTG​GCC​AAG​TA	TGG​GAC​AAA​TCT​TCA​GGT​TCC​A	92
*TBP*	TATA-binding protein (housekeeper)	CAA​GCG​GTT​TGC​TGC​TGT​AA	CAC​CAT​CTT​CCC​GGA​ACT​GAA	79
*UBE2D2*	Ubiquitin Conjugating Enzyme E2 D2 (housekeeper)	CAG​TGC​TGC​GTG​TTG​TAC​ATA	TGC​TAG​GAG​GCA​ATG​TTG​GTA	76
*VEGFA*	Vascular endothelial growth factor A	GGA​GAA​TGT​CCC​TCC​CAG​AA	GCC​TCC​CTA​GAA​GGG​ACA​AA	84
*YWHAZ*	Tyrosine 3-Monooxygenase (housekeeper)	GCT​TCA​CAA​GCA​GAG​AGC​AA	CAG​CAA​CTT​CGG​CCA​AGT​AA	76

Primer sequences for detection of genes of interest in the guinea pig hippocampus. Primer sequences are displayed from 5’-3’ for forward and reverse primers.

### Statistical Analyses

Statistical analyses were programmed using SAS v9.4 (SAS Institute, Cary, North Carolina, United States). A priori, *p* < 0.05 (two-tailed) was used to indicate statistical significance. Graphs were made using Prism v9.0 (Graphpad Software Inc., La Jolla, California) and data presented as adjusted means ± SEM. For outcomes that were measured over time, the difference between untreated preterm animals and those receiving LOW-, MID- and HIGH-GNX was examined with the inclusion of fixed effects for time and the interaction of treatment and time, and random intercepts and random slopes for pups nested within dam*.* For outcomes that were measured at one time, mixed linear regression modelling with comparisons made between term and all preterm groups with random intercepts and random slopes for the effect of dam included in the model. Assumptions for mixed linear regression were examined and were deemed appropriate.

## Results

### Physical Characteristics During Preterm Period

All pups born preterm were extensively monitored until they reached term equivalence age (7 days since birth). Key indicators of health and wellbeing included daily fractional weight gain (Figures 1A,B), ponderal index as weight (kg)/length (m^3^) (Figures 1C,D), daily supplemental feeding volume (Figures 1E,F), and a wellbeing score taken at 2 h post GNX dose or at the equivalent time for controls (Figures 1G,H). High dose ganaxolone significantly impaired fractional weight gain for males compared to preterm control males (postnatal day 2: *p* = 0.002; 3: *p* = 0.005; 4: *p* = 0.003; 5: *p* < 0.001; 6: *p* < 0.001; and 7: *p* = 0.001). Ganaxolone also impaired fractional weight gain for female preterm neonates at postnatal day 2 (low *p* = 0.049; mid *p* = 0.024; high *p* < 0.001), 3 (low *p* = 0.026; high *p* < 0.001), 4 (high *p* = 0.012), 5 (high *p* = 0.016), 6 (high *p* = 0.016), and term equivalence (7; high *p* = 0.016). Ponderal index was unaffected for females receiving ganaxolone, however males receiving high dose had a significantly lower ponderal index than preterm control males at postnatal day 4 (*p* = 0.044). Preterm males receiving mid and high dose ganaxolone required significantly larger volumes of supplemental feeding at postnatal day 3 (mid *p* = 0.022; high *p* = 0.007), whilst females receiving high dose required significantly more at postnatal day 1 (*p* = 0.009) and 2 (*p* = 0.001). Whilst wellbeing score was lower for males receiving ganaxolone on their day of birth (mid *p* = 0.023; high *p* = 0.001) and postnatal day 1 (high *p* = 0.046), females receiving ganaxolone appeared to be impacted to a greater degree, achieving lower wellbeing scores for longer (day of birth: high *p* = 0.019; postnatal day 1: low *p* = 0.014; mid *p* < 0.001; high *p* < 0.001; 2: mid *p* = 0.002; high *p* < 0.001; 3: mid *p* = 0.015; high *p* < 0.001; and 4: high *p* < 0.001) compared to the control preterm neonates. There were no other significant differences identified. Adjusted means, SEM and model *p*-value for each parameter are available in the supplemental text.

### Body Measurements and Organ Weights at Term Equivalence

At the time of tissue collection (term equivalence) body and organ weights (Table 3), in addition to body measurements (Table 4) were recorded and are displayed below. There was no significant effect of ganaxolone on body weights for animals born preterm of either sex when compared to their term-born counterparts. Similarly, there were also no significant differences identified for absolute brain, hippocampus, ½ cerebellum, liver, or heart weights. However, ganaxolone treatment appeared to affect kidney and adrenal weights for males. Kidneys of preterm males receiving low (*p* = 0.001), mid (*p* = 0.001) and high (*p* = 0.004) dose were significantly heavier than those of term males. Adrenals of preterm males receiving low (*p* = 0.035), mid (*p* = 0.010), and high (*p* = 0.017) dose were also heavier than term males. Interestingly, this increase in weight appears to be due to the vehicle, as the kidneys (males *p* = 0.0023; females *p* = 0.032) and adrenals (males *p* = 0.012) of vehicle-treated preterm neonates were significantly heavier than untreated preterm controls (Supplementary Table S7). There was no significant difference between male preterm controls and term-born for either kidney or adrenal weight, or between any of the female preterm groups. Visceral fat was significantly reduced in all preterm groups compared to term (males: all *p* < 0.001; females: control *p* = 0.002; low *p* = 0.002; mid *p* = 0.002; and high *p* < 0.001). Conversely, subcutaneous fat was only lighter for control preterm males (*p* = 0.010) and those treated with high dose ganaxolone (*p* = 0.047) when compared to male term-born controls. Body measurements were largely unaffected by timing of delivery, apart from hock toe length which was significantly shorter for all preterm males compared to term (control *p* = 0.010; low = 0.023; mid = 0.023; high *p* = 0.009). There were no other significant anthropometric differences identified, including between vehicle-treated and untreated control preterm neonates (Supplementary Table S7).

**TABLE 3:** Body and organ weights at term equivalence.

Sex	Delivery	Treatment	Wgt 0	Brain Wgt	Hippo. Wgt	Cereb. Wgt	Liver Wgt	Heart Wgt	Kidney Wgt	Adrenals Wgt	Sub. Cut. Fat Wgt	Visc. Fat Wgt
Male	Term		97.2 ± 2.7	2.61 ± 0.05	0.112 ± 0.004	0.141 ± 0.005	3.97 ± 0.27	0.395 ± 0.018	0.83 ± 0.027	0.034 ± 0.002	1.40 ± 0.06	0.837 ± 0.037
	Preterm	—	90.8 ± 7.2	2.46 ± 0.08	0.116 ± 0.004	0.158 ± 0.015	2.92 ± 0.24	0.320 ± 0.023	0.94 ± 0.073	0.034 ± 0.002	0.90 ± 0.13*	0.323 ± 0.060*
		Low-GNX	95.1 ± 3.3	2.43 ± 0.04	0.109 ± 0.007	0.150 ± 0.008	3.65 ± 0.25	0.336 ± 0.019	1.31 ± 0.054*	0.044 ± 0.003*	1.22 ± 0.11	0.331 ± 0.038*
		Mid-GNX	93.3 ± 3.8	2.47 ± 0.03	0.112 ± 0.006	0.151 ± 0.009	3.53 ± 0.30	0.331 ± 0.011	1.35 ± 0.026*	0.048 ± 0.001*	1.11 ± 0.12	0.310 ± 0.049*
		High-GNX	84.0 ± 6.8	2.43 ± 0.05	0.103 ± 0.007	0.148 ± 0.015	3.02 ± 0.21	0.321 ± 0.020	1.22 ± 0.085*	0.046 ± 0.003*	0.89 ± 0.13*	0.288 ± 0.056*
Female	Term		98.6 ± 4.3	2.52 ± 0.04	0.123 ± 0.007	0.158 ± 0.010	3.91 ± 0.29	0.386 ± 0.024	0.85 ± 0.045	0.037 ± 0.005	1.22 ± 0.09	0.663 ± 0.050
	Preterm	—	88.4 ± 5.8	2.37 ± 0.04	0.097 ± 0.004	0.142 ± 0.007	3.17 ± 0.34	0.304 ± 0.027	0.98 ± 0.081	0.039 ± 0.002	0.79 ± 0.12	0.288 ± 0.056*
		Low-GNX	86.4 ± 5.0	2.39 ± 0.07	0.108 ± 0.007	0.161 ± 0.010	2.95 ± 0.26	0.300 ± 0.018	1.24 ± 0.072	0.049 ± 0.002	0.90 ± 0.07	0.230 ± 0.029*
		Mid-GNX	80.7 ± 3.1	2.34 ± 0.04	0.108 ± 0.007	0.160 ± 0.007	2.88 ± 0.16	0.268 ± 0.008	1.14 ± 0.066	0.047 ± 0.002	0.81 ± 0.05	0.243 ± 0.021*
		High-GNX	77.5 ± 9.0	2.27 ± 0.09	0.105 ± 0.009	0.141 ± 0.009	2.75 ± 0.49	0.273 ± 0.027	1.18 ± 0.117	0.049 ± 0.004	0.74 ± 0.16	0.234 ± 0.047*

Means and standard error of the means for body and organ weights (in grams) recorded upon tissue collection at term equivalence age. All weights are in grams. Comparisons are made within sex with difference to term at *p* < 0.05 signified by *. GNX, ganaxolone, wgt = weight, hippo = hippocampus, cereb = cerebellum, sub cut = subcutaneous, visc = visceral.

**TABLE 4:** Body measurements at term equivalence.

Sex	Delivery	Treatment	Nose-rump	Head Length	Crown rump	Hind limb	Hock Toe	Head Circ	Neck Circ	Chest Circ	Abdo. Circ
Male	Term		168.6 ± 2.4	53.1 ± 2.4	130.9 ± 1.8	38.0 ± 0.8	37.3 ± 0.5	87.8 ± 0.8	74.9 ± 1.3	92.6 ± 2.5	96.6 ± 2.6
	Preterm	—	158.6 ± 3.4	48.6 ± 2.3	127.0 ± 3.2	35.9 ± 0.8	34.6 ± 0.6*	88.8 ± 2.9	70.3 ± 2.5	90.0 ± 3.4	94.9 ± 4.2
		Low-GNX	163.8 ± 2.1	51.2 ± 2.3	130.2 ± 1.6	36.3 ± 0.4	35.0 ± 0.4*	85.7 ± 1.3	69.7 ± 1.2	90.2 ± 1.9	97.2 ± 3.2
		Mid-GNX	163.7 ± 1.5	49.7 ± 1.8	129.5 ± 2.5	36.3 ± 0.6	35.0 ± 0.4*	86.5 ± 1.6	70.3 ± 2.9	92.8 ± 1.9	96.7 ± 3.6
		High-GNX	157.5 ± 4.7	48.0 ± 1.2	126.3 ± 3.7	35.3 ± 0.4	34.3 ± 0.6*	84.8 ± 2.8	66.7 ± 2.7	89.3 ± 3.8	93.5 ± 5.3
Female	Term		168.4 ± 2.4	53.4 ± 1.6	131.6 ± 2.3	38.3 ± 0.6	37.3 ± 0.5	88.9 ± 1.2	74.7 ± 1.6	90.4 ± 1.7	94.9 ± 2.7
	Preterm	—	158.9 ± 2.8	47.6 ± 1.2	126.9 ± 3.0	36.0 ± 0.7	34.6 ± 0.6	85.3 ± 1.1	68.3 ± 4.3	89.3 ± 2.3	93.6 ± 3.5
		Low-GNX	159.5 ± 3.1	46.5 ± 2.2	125.8 ± 1.9	35.8 ± 0.7	34.3 ± 0.6	83.8 ± 1.2	66.7 ± 1.8	88.7 ± 2.5	92.5 ± 3.3
		Mid-GNX	157.5 ± 2.1	51.5 ± 2.2	122.5 ± 2.3	35.3 ± 0.6	34.2 ± 0.5	82.0 ± 1.1	68.2 ± 2.8	85.3 ± 1.7	90.2 ± 3.5
		High-GNX	154.6 ± 5.4	46.6 ± 1.9	119.8 ± 5.6	34.7 ± 1.0	33.8 ± 1.1	81.8 ± 2.1	65.4 ± 2.4	86.8 ± 5.0	92.0 ± 5.8

Means and standard error of the means for body measurements (in millimetres) recorded upon tissue collection at term equivalence age. Comparisons are made within sex with difference to term at *p* < 0.05 signified by *. GNX, ganaxolone, circ = circumference, abdo = abdominal.

### Myelination and Neuronal Nuclei Protein Expression in the Hippocampus and Subcortical White Matter

**FIGURE 2:**
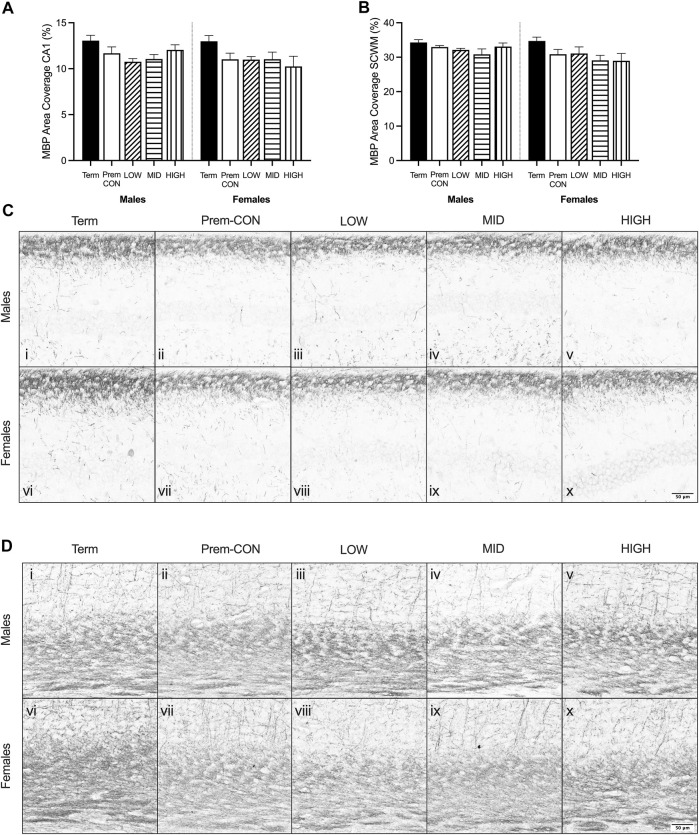
Myelin basic protein (MBP) immunostaining was quantified in the brain of term control (black; males *n* = 8, females *n* = 7), preterm control (white; males *n* = 7, females *n* = 7), low-GNX (diagonal stripes; males *n* = 6, females *n* = 5), mid-GNX (horizontal stripes; males *n* = 6, females *n* = 6), and high-GNX (vertical stripes; males *n* = 6, females *n* = 5) for **(A)** the CA1 region of the hippocampus and **(B)** the overlying subcortical white matter. Data presented as means ± SEM with * indicating significance at *p* < 0.05. **(C)** Representative photomicrographs of MBP immunostaining of the **(C)** CA1 region and **(D)** overlying subcortical white matter (i, vi = term; ii, vii = preterm control; iii, viii = low-GNX preterm; iv, ix = mid-GNX preterm; v, x = high-GNX preterm). Scale bar = 50 μm.

**FIGURE 3:**
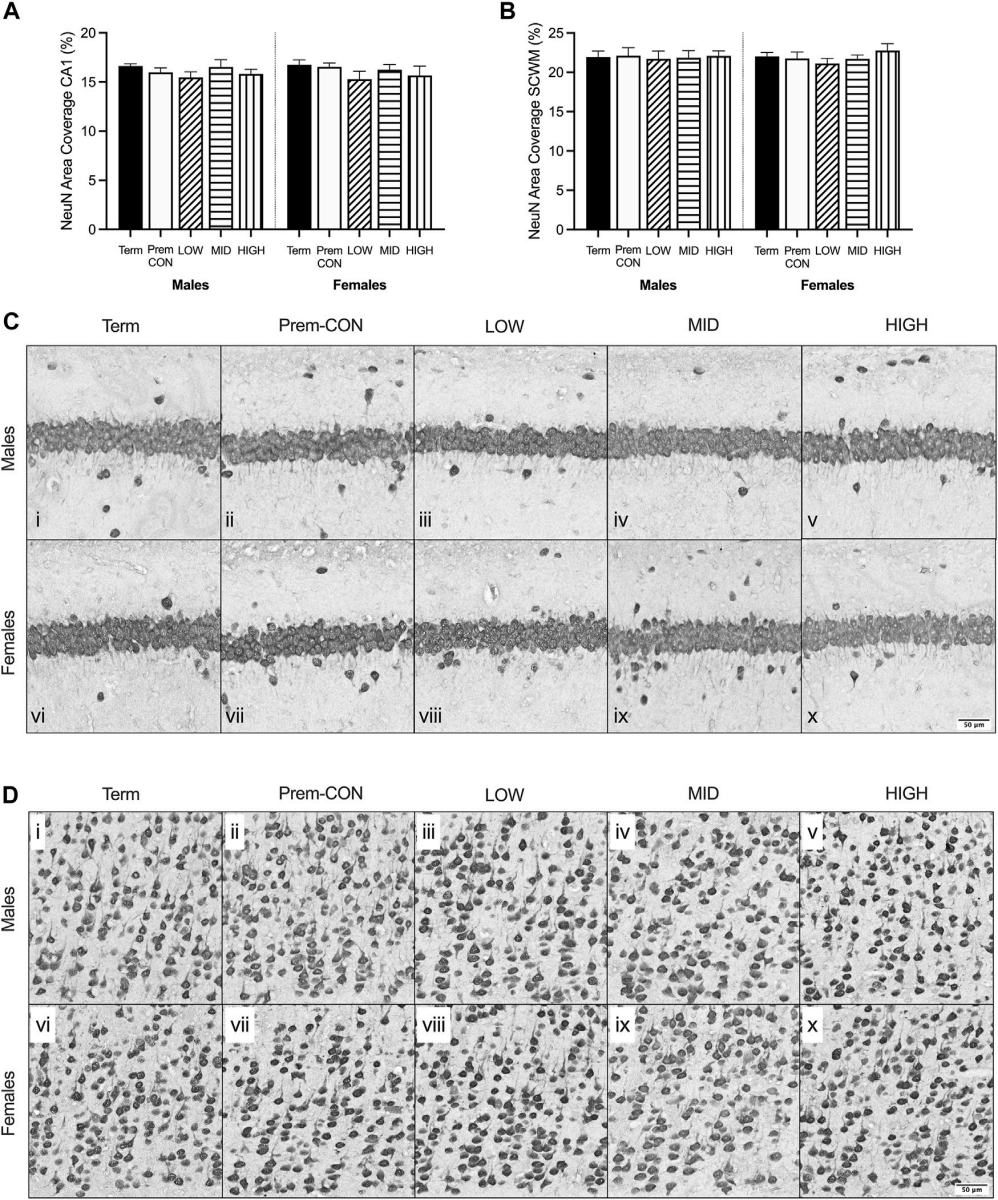
Neuronal nuclei (NeuN) immunostaining was quantified in the brain of term control (black; males *n* = 8, females *n* = 8), preterm control (white; males *n* = 6, females *n* = 7), low-GNX (diagonal stripes; males *n* = 6, females *n* = 5), mid-GNX (horizontal stripes; males *n* = 6, females *n* = 5), and high-GNX (vertical stripes; males *n* = 6, females *n* = 5) for **(A)** the CA1 region of the hippocampus and **(B)** the overlying subcortical white matter. Data presented as means ± SEM with * indicating significance at *p* < 0.05. **(C)** Representative photomicrographs of NeuN immunostaining of the **(C)** CA1 region and **(D)** overlying subcortical white matter (i, vi = term; ii, vii = preterm control; iii, viii = low-GNX preterm; iv, ix = mid-GNX preterm; v, x = high-GNX preterm). Scale bar = 50 μm.

Mature myelin (Figure 2) and neuronal nuclei (Figure 3) protein area coverage was quantified in the CA1 region of the hippocampus and overlying subcortical white matter by immunohistochemistry. There were no significant differences identified between the preterm groups and the term controls for either sex. Adjusted means, SEM and model *p*-value for each parameter are available in the supplemental text.

### Relative mRNA Expression of Oligodendrocyte Lineage and Neuron Markers

**FIGURE 4:**
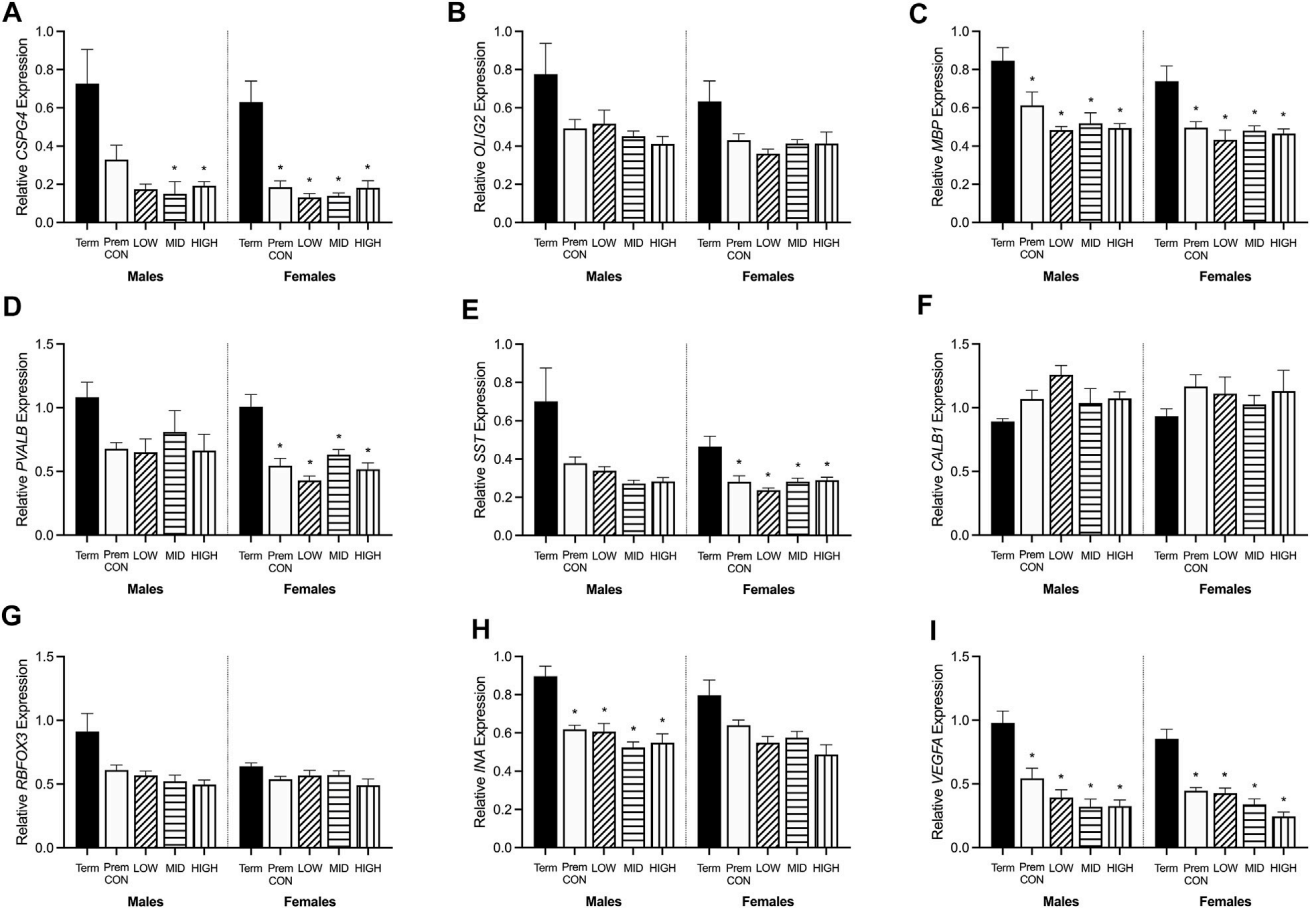
Relative mRNA expression for oligodendrocyte and neuronal markers in the hippocampus of term control (black; males *n* = 8, females *n* = 7), preterm control (white; males *n* = 7, females *n* = 6), low-GNX (diagonal stripes; males *n* = 5, females *n* = 6), mid-GNX (horizontal stripes; males *n* = 6, females *n* = 6), and high-GNX (vertical stripes; males *n* = 6, females *n* = 6). **(A)** *CSPG4*, **(B)** *OLIG2*, **(C)** *MBP*, **(D)** *PVALB*, **(E)** *SST*, **(F)** *CALB1*, **(G)** *RBFOX3*, **(H)** *INA*, and **(I)** *VEGFA*. Data presented as means ± SEM with * indicating significance at *p* < 0.05.

Hippocampal mRNA expression of key oligodendrocyte lineage and neuron development genes were quantified by high throughput Fluidigm and are displayed in Figure 4. Markers of early stage (*CSPG4*, Figure 4A) and late stage (*MBP*, Figure 4C) cells of the oligodendrocyte lineage were significantly higher in term cohorts compared to preterm. *CSPG4* expression was significantly higher in term males compared to those receiving mid (*p* = 0.045) and high (*p* = 0.05) dose ganaxolone, and in females the expression in term-born animals was significantly higher than in all preterm groups (control *p* = 0.006; low *p* = 0.004; mid *p* = 0.005; high *p* = 0.010). *MBP* expression was also significantly higher in term males compared to all preterm groups (control *p* = 0.029; low *p* = 0.019; mid *p* = 0.010; high *p* = 0.010), and in term females compared to all preterm groups (control *p* = 0.019; low *p* = 0.008; mid *p* = 0.015; high 0.015). Meanwhile, the expression of *OLIG2* (Figure 4B, representing the entire oligodendrocyte lineage) was not significantly higher in the term cohorts compared to preterm. Markers of GABAergic interneurons were also significantly decreased in preterm females with *PVALB* (parvalbumin, Figures 4D) and SST (somatostatin, Figure 4E) significantly lower in all preterm female groups compared to term (*PVALB*: control *p* = 0.004; low *p* = 0.001; mid *p* = 0.006; high *p* = 0.002; *SST*: control *p* = 0.010; low *p* = 0.004; mid *p* = 0.010; high *p* = 0.015). There was also a statistically nonsignificant trend in males for both markers. Conversely, neither *CALB1* (calbindin, Figure 4F) nor *RBFOX3* (NeuN, Figure 4G) were significantly affected by preterm birth or treatment in either sex. Interestingly, neuron assembly appears to be impacted by preterm delivery with *INA* (alpha-internexin, Figure 4H) significantly decreased in all preterm male groups compared to term (control *p* = 0.007; low *p* = 0.008; mid *p* = 0.003; high *p* = 0.004). There was also a statistically non-significant trend in females. Overall cell migration and growth as quantified by *VEGFA* expression (Figure 4I), was also significantly decreased in all preterm groups compared to term-born animals (males: control *p* = 0.010; low *p* = 0.005; mid *p* = 0.003; high *p* = 0.003; females: control *p* = 0.001; low *p* = 0.002; mid *p* < 0.001; high *p* < 0.001). There were no other significant differences identified. Adjusted means, SEM and model *p*-value for each parameter are available in the supplemental text.

### Relative Expression of GABA and Glutamate Synthetic Enzymes, Transporters, and Receptors

**FIGURE 5:**
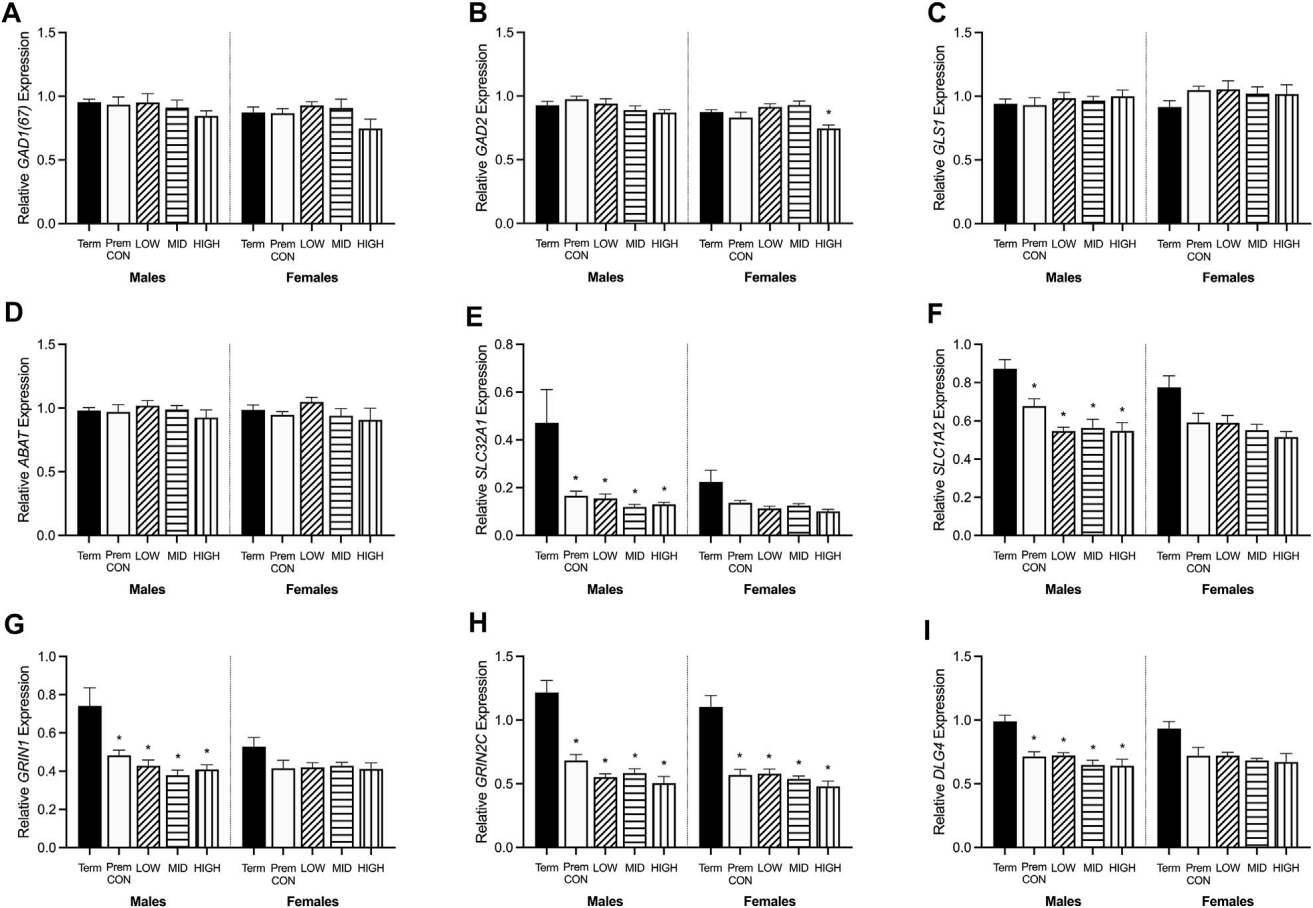
Relative mRNA expression for GABA and glutamatergic markers in the hippocampus of term control (black; males *n* = 8, females *n* = 7), preterm control (white; males *n* = 7, females *n* = 6), low-GNX (diagonal stripes; males *n* = 5, females *n* = 6), mid-GNX (horizontal stripes; males *n* = 6, females *n* = 6), and high-GNX (vertical stripes; males *n* = 6, females *n* = 6). **(A)** *GAD(67)*, **(B)** *GAD2*, **(C)** *GLS1*, **(D)** *ABAT*, **(E)** *SLC32A1*, **(F)** *SLC1A2*, **(G)** *GRIN1*, **(H)** *GRIN2C*, and **(I)** *DLG4*. Data presented as means ± SEM with * indicating significance at *p* < 0.05.

**FIGURE 6:**
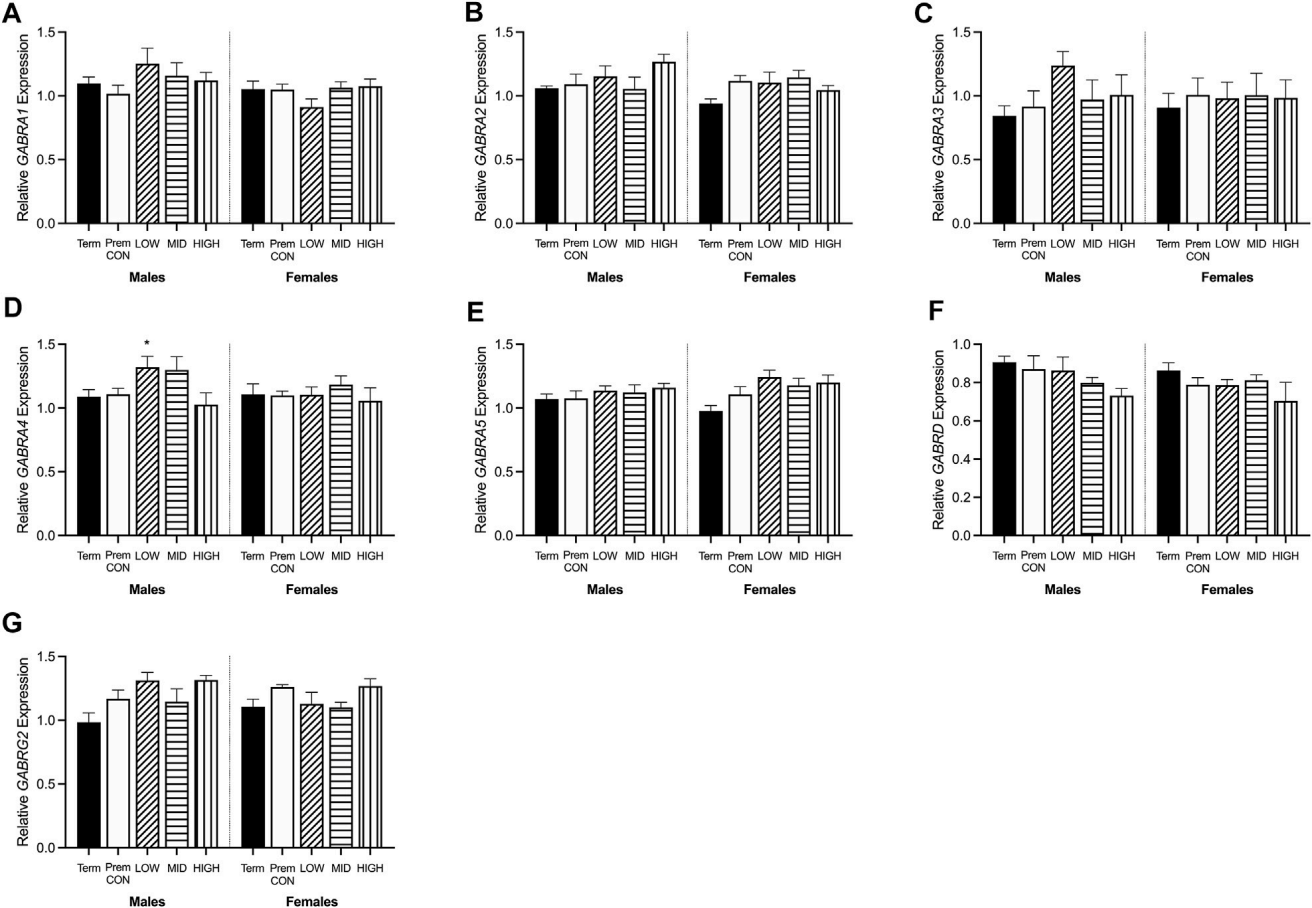
Relative mRNA expression for GABAA receptors in the hippocampus of term control (black; males *n* = 8, females *n* = 7), preterm control (white; males *n* = 7, females *n* = 6), low-GNX (diagonal stripes; males *n* = 5, females *n* = 6), mid-GNX (horizontal stripes; males *n* = 6, females *n* = 6), and high-GNX (vertical stripes; males *n* = 6, females *n* = 6). **(A)** *GABRA1*, **(B)** *GABRA2*, **(C)** *GABRA3*, **(D)** *GABRA4*, **(E)** *GABRA5*, **(F)** *GABRD*, and **(G)** *GABRG2*. Data presented as means ± SEM with * indicating significance at *p* < 0.05.

Hippocampal mRNA expression of key GABA and glutamate synthetic enzyme, transporter and receptor genes were quantified by high throughput Fluidigm and are displayed in Figures 5, 6.

GABA and glutamate synthetic enzymes [*GAD1(67)*, *GAD2*, *GLS1*, and *ABAT*, Figures 5A–D] were largely unaffected by preterm birth, apart from a significant decrease in *GAD2* expression in the preterm females receiving high dose ganaxolone (*p* = 0.029). However, transport of GABA into synaptic vesicles (*SLC32A1*; vGAT, Figure 5E) and reuptake of synaptic glutamate (*SLC1A2*; EAAT2, Figure 5F) were significantly decreased in all male preterm groups compared to term born animals (*SLC32A1*: control *p* = 0.018; low *p* = 0.048; mid *p* = 0.030; high *p* = 0.037; *SLC1A2*: control *p* = 0.018; low *p* = 0.007; mid *p* = 0.006; high *p* = 0.006). There was also a statistically non-significant trend towards a reduction in females. Additionally, key glutamate receptors (*GRIN1* and *GRIN2C*, Figures 5G,H) which are involved in memory and learning through their interactions with *DLG4* (post-synaptic density protein, Figure 5I) were also significantly decreased in all preterm male groups (*GRIN1*: control *p* = 0.027; low *p* = 0.021; mid *p* = 0.009; high *p* = 0.014; *GRIN2C*: control *p* = 0.003; low *p* = 0.002; mid *p* < 0.001; high *p* < 0.001; *DLG4*: control *p* = 0.008; low *p* = 0.007; mid *p* = 0.002; high *p* = 0.002) compared to the term-born males. This also appeared to be the case for preterm females but only reached significance for *GRIN2C* (control *p* < 0.001; low *p* < 0.001; mid *p* < 0.001; high *p* < 0.001). GABA_A_ receptors (Figure 6) in the hippocampus were largely unaffected by preterm delivery or treatment, with only males receiving low ganaxolone dose showing a significant increase in *GABRA4* expression compared to term males (Figure 6D, *p* = 0.027). There were no other significant differences identified. Adjusted means, SEM and model *p*-value for each parameter are available in the supplemental text.

## Discussion

The aim of this study was to identify the lowest safe and effective dose of ganaxolone for improving brain development following preterm birth. Our previous studies show that 5 mg/kg daily from birth until term equivalence in the guinea pig was able to ameliorate behavioural changes and improve myelination at the equivalent of childhood in male guinea pigs born preterm (Shaw et al., 2019). Despite these promising results, there were adverse side effects during the treatment period that necessitated revisiting the dosing regimen. In the present study three lower doses were used (0.25, 0.5, and 1.25 mg/kg twice daily), and a particular focus placed on the physical effects within the treatment period. Of the three doses, as expected it was the highest dose that invoked the greatest effects on physical wellbeing. For both male and female preterm neonates, daily fractional weight gain was largely reduced in those receiving the high dose, suggesting a reduction in level of activity that prevented active suckling from their mother. Ponderal index was also decreased in males receiving the high dose, again highlighting the altered growth trajectory associated with the higher dose. Following on from these results it is not unexpected that neonates receiving the high dose also required additional supplemental feeding, but this was still insufficient at replacing nutritional support from the dam as evidenced by the reduced weight gain in these neonates. An aggregate wellbeing score was also recorded each day following the treatment doses, and represented level of respiration, movement, and posture. Interestingly, it was females that were affected to a larger degree here with lower aggregate scores across the treatment period for mid and high doses, whilst males were only affected in the early postnatal period. This score is an effective representation of activity level, and whilst some sedation during this period may be beneficial for brain development by mimicking the ‘fetal sleep state’ (Nicol et al., 2001; Hirst et al., 2008), a reduced activity level may lead to a reduction in overall growth due to reduced independent feeding, as seen for the higher dose. Altogether, these findings suggest that in the clinical setting a neonate receiving this level of ganaxolone therapy may require additional support. Importantly, neonates receiving mid and low dose ganaxolone did not have these physical side-effects to the extent nor the duration of those receiving the high dose suggesting that long term studies, similar to the original pilot study, should be performed to examine functional behavioural outcomes using these lower treatment doses.

Whilst we are unable to compare these physical parameters to a fetus of the same post-conceptional age, body measurements and organ weights at term equivalence can reveal some information on growth of treated and untreated preterm neonates compared to appropriately grown term neonates. After a week of *ex utero* growth, the preterm neonates were no different to those born at term (regardless of treatment) for body measurements apart from males have a lesser hock-toe length. Absolute organ weights however were altered by preterm delivery and treatment, once again highlighting how basic anthropometric measures may not capture important changes in growth at a tissue level despite this being critical for optimal long-term growth and multi-system wellbeing. For instance, visceral fat was reduced for all preterm groups suggesting this is a result of prematurity as fat is laid down in the last trimester. Other organ systems appeared to be differentially affected by treatment rather than by gestational age; adrenal and kidney weights were increased for males receiving ganaxolone suggesting a treatment effect. However, upon further investigation, it was revealed that this effect could be attributed to the vehicle. In future studies utilising this vehicle, renal analysis should be included to determine the impact on kidney and adrenal development.

When looking at the brains of these neonates, it appears that myelin protein is unaffected. However we know from previous studies that at 24 h after birth, myelination of the preterm brain is reduced compared to 24 h after term birth (Timby et al., 2006). We also know that at the equivalent of childhood age there is a reduction in myelination of the preterm brain compared to age-matched term brain (Shaw et al., 2016; Shaw et al., 2019). Thus, the findings here suggest that there is a period of catch-up growth for myelination until at least term equivalence, which fails to continue along an optimal trajectory after TEA. The relative mRNA expression may hold the key to this failure in ongoing development, with reductions in early (*CSPG4*, also known as NG2) and final stage oligodendrocyte maturation (*MBP*) seen in preterm neonates. A reduction in the transcription of this mRNA at both ends of the oligodendrocyte lineage may set up for an ongoing reduction in protein translation, ultimately resulting in reduced myelination later in life. Unfortunately, none of the ganaxolone doses were able to reverse this effect and thus may not have been sufficient (dose itself, dose frequency or duration of treatment) to prevent long-term deficits. Prominent GABAergic interneurons within the hippocampus (*PVALB*, *SST*) were also reduced in preterm neonates, regardless of treatment, which may also play into the hypothesised lack of ongoing catch-up myelination. Oligodendrocytes and GABAergic interneurons are tightly coupled in terms of development and function, with the maturation of oligodendrocytes promoted by interaction with GABAergic interneurons, and the function of GABAergic interneurons promoted by myelin sheathing (Boulanger and Messier, 2017; Micheva et al., 2018). It is important to note that all preterm neonates in the study received a single course of betamethasone (48 and 24 h prior to birth) to improve lung function and ultimately, survival. This may have dampened the effect of ganaxolone, as without betamethasone a greater degree of brain injury, particularly hypoxic oligodendrocyte injury, may have been seen for which ganaxolone therapy may have had a greater benefit. Neurons overall don’t appear to be affected in the current model of preterm birth, with no differences observed in protein or mRNA (*RBFOX3*), however the gene responsible for the assembly of neurons is reduced in preterm neonates (*INA*), in addition to a marker of overall growth (*VEGFA*). These two key genes may also be contributing to the lack of GABAergic interneuron mRNA and emphasises the immature nature of the preterm brain.

Ganaxolone, like allopregnanolone, exerts its inhibitory effects on the central nervous system through binding to extrasynaptic GABA_A_ receptors (Nohria and Giller, 2007; Lambert et al., 2009). We have previously shown in the cerebellum that the delta subunit exhibits a dramatic increase in expression in the term-born neonate compared to a preterm neonate at 24 h of age; this may be an adaptation to decreased levels of allopregnanolone following birth that does not occur in the preterm cerebellum. The same does not occur in the hippocampus, possibly due to slightly differing timelines for development. Here, there were again no stark differences between the term and term equivalence preterm neonate. However, some of the extrasynaptic GABA_A_ receptor subunits appear to increase in response to ganaxolone, particularly in the males, and with additional numbers these trends may prove significant suggesting a positive feedback mechanism in response to ganaxolone.

By positively modulating the GABA_A_ receptors, ganaxolone (and allopregnanolone) increase the binding of GABA, thus raising inhibitory tone in the brain (Nohria and Giller, 2007; Lambert et al., 2009). There is a growing number of studies suggesting that preterm birth may disrupt the GABAergic pathway and result in a shift towards excitation due to a dominance of glutamatergic transmission (Sánchez-Gómez et al., 2003; Robinson et al., 2006; Shaw et al., 2018; Basu et al., 2020; Shaw et al., 2021). Whilst we are unable to comment on levels of GABA and glutamate in our preterm neonates, we did measure mRNA responsible for the production and breakdown of GABA and glutamate. At TEA there were no major differences between the term and preterm neonates for any of these genes (*GAD67(1)*, *GAD2*, *GLS1*, *ABAT*) suggesting that production and breakdown are not impacted by preterm delivery by the time the offspring had reached term equivalence. The highest dose of ganaxolone did decrease the expression of *GAD2* in females, which may represent a negative feedback mechanism whereby additional GABA is not required due to the high presence of ganaxolone occupying GABA_A_ receptors. Where there are apparent differences between the term and preterm neonate (regardless of treatment) is the transport of GABA (*SLC32A1*, also known as vGAT) and glutamate (*SLC1A2*, also known as EAAT2). Reduced expression of vGAT (vesicular inhibitory amino acid transporter) may represent an adaptive mechanism in preterm neonates to increase the amount of available GABA within the synapse for maintaining inhibitory action. However, the reduced expression of EAAT2 (excitatory amino acid transporter 2) indicates that glutamate clearance from the synapse may be limited. This in turn may be explained, at least in part, by the decreased expression of key glutamate NMDA receptors (*GRIN1*, *GRIN2D*) involved in formation of memory and synaptic plasticity (Chen et al., 2009; Wei et al., 2016) through interaction with *DLG4* (also known as PSD-95) (Kim and Sheng, 2004). A reduction in these genes is hypothesised to result in learning disorders (Chen et al., 2009; Wei et al., 2016), which is a common sequelae in survivors of preterm birth.

In conclusion, based on the neuroprotection demonstrated in our pilot work the purpose of the current study was to investigate the impact of a dosing range on tolerability and sedation in male and females born preterm to inform future longer-term studies on functional outcomes. We have shown that low and mid doses of ganaxolone only resulted in short-term side effects within the preterm neonatal treatment period suggesting that the therapy was being administered at an effective level with increased tolerability and safety compared to the high dose which significantly impaired weight gain in guinea pig pups born preterm. The longer-term effects of reduced activity and growth therefore require further studies although the current data support the use of the lower doses in ongoing studies. Whilst we have not identified improvements in key proteins and genes in response to ganaxolone, it may be too early for some of these effects to be apparent as our previous data does suggest a clear improvement in myelination and behaviour at the equivalence of childhood. Therefore, long-term behavioural studies using the mid should be performed and will investigate any sex-specific dosing requirements. Finally, we have identified additional stark alterations in oligodendrocyte, neuronal, GABAergic, and glutamatergic genes in preterm neonates that may play integral roles in developmental programming for poor outcomes in preterm born offspring and warrants further investigation within this area.

## Data Availability

The original contributions presented in the study are included in the article/Supplementary Material, further inquiries can be directed to the corresponding author.

## Supplementary Material

Supplementary Figure S1 Preterm survival from birth until term equivalence (7 days since birth) for **(A)** males and **(B)** females receiving no treatment (control), vehicle (45% b-cyclodextrin), 0.5 mg/kg ganaxolone (low dose), 1.0 mg/kg ganaxolone (mid dose), or 2.5 mg/kg ganaxolone (high dose). Group numbers at birth to TEA were control (males = 11–7; females = 9–7), vehicle (males = 11–6; females = 8–6), low (males = 8–6; females = 7–6), mid (males = 9–6; females = 9–6), and high (males = 8–6; females = 10–5).

Supplementary Figure S2 Fractional weight gain **(A,B)**, ponderal index **(C,D)**, supplemental feeding **(E,F)**, and wellbeing score **(G,H)** was recorded from birth (days since birth 0) until term equivalence (postnatal age 7) for male **(A,C,E,G)** and female **(B,D,F,H)** preterm neonates receiving vehicle (45% β-cyclodextrin; diamond; males *n* = 6, females *n* = 6) and compared to control (circle; males *n* = 7, females *n* = 7) preterm neonates. Data presented as means ± SEM.
