# Supplementary figures and images for: Examining Neurosteroid-Analogue Therapy in the Preterm Neonate For Promoting Hippocampal Neurodevelopment
Source: Front Physiol. 2022 Apr 19;13:871265. doi: 10.3389/fphys.2022.871265 (PMC9062084; doi:10.3389/fphys.2022.871265)

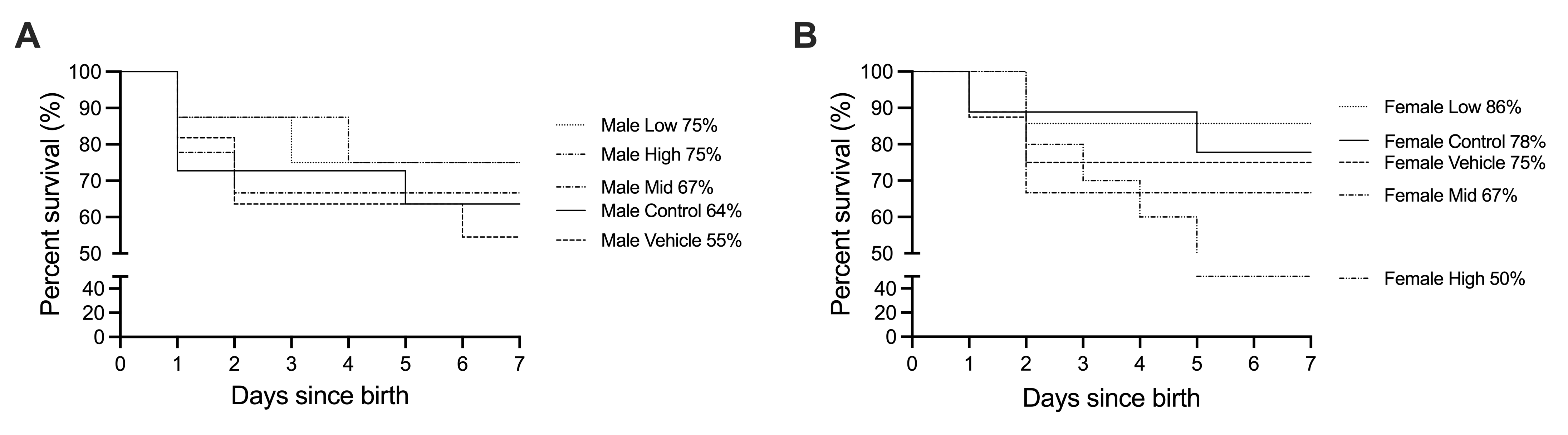

Supplement: Supplementary file 1 [file Image1.TIFF]

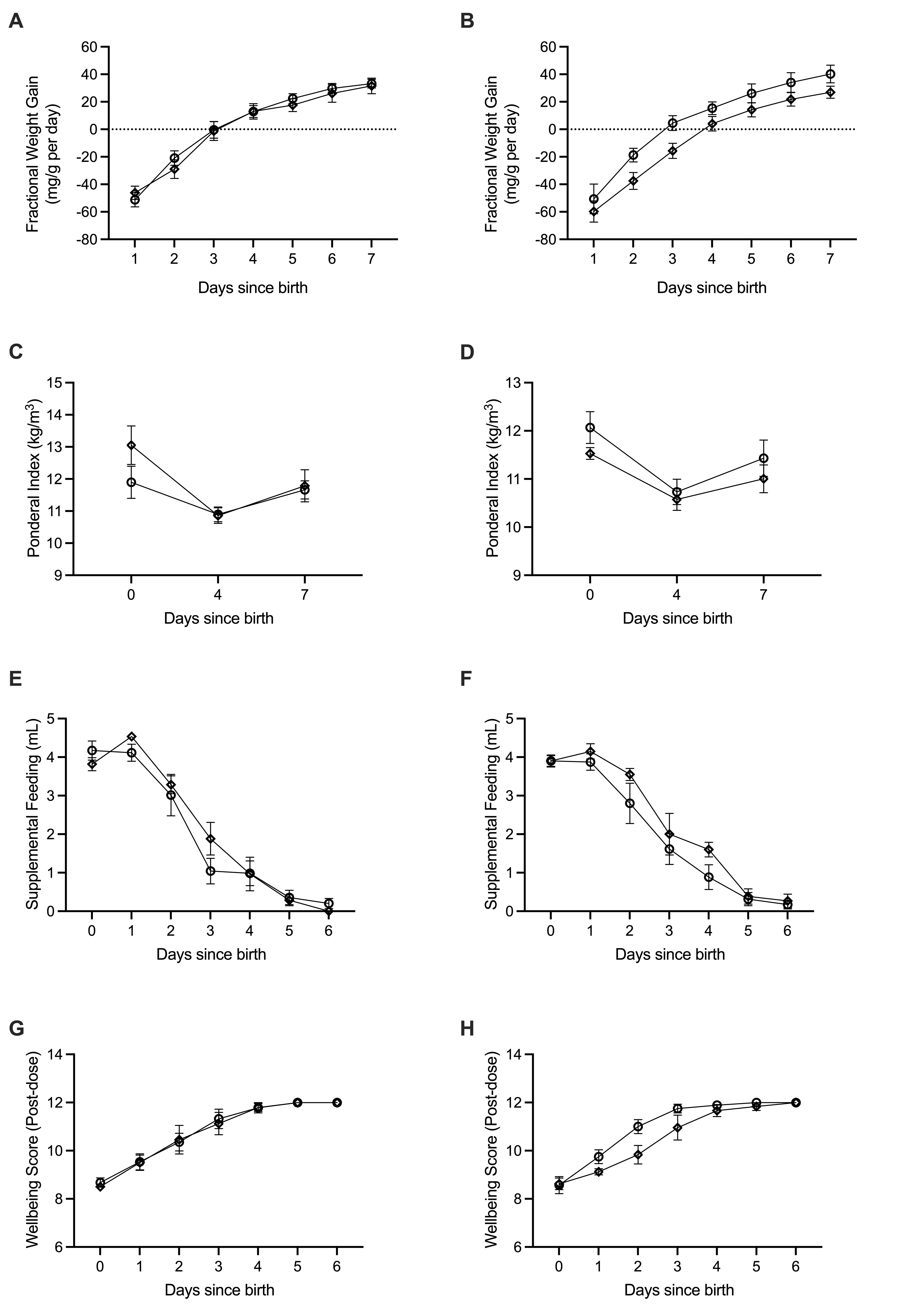

Supplement: Supplementary file 2 [file Image2.TIFF]
