# Supplementary material for: Examining Neurosteroid-Analogue Therapy in the Preterm Neonate For Promoting Hippocampal Neurodevelopment
Source: Front Physiol. 2022 Apr 19;13:871265. doi: 10.3389/fphys.2022.871265 (PMC9062084; doi:10.3389/fphys.2022.871265)
Supplement: Supplementary file 3 [file DataSheet1.docx]

Supplementary Tables:

ST1. Fractional weight gain (adjusted means and SEM)

| Sex | Treatment | 1 | 2 | 3 | 4 | 5 | 6 | 7 | Model p-value |
| --- | --- | --- | --- | --- | --- | --- | --- | --- | --- |
| Male | - | -51.26 ± 4.7 | -20.91 ± 4.9 | -0.37 ± 5.6 | 12.92 ± 4.0 | 22.33 ± 3.2 | 29.73 ± 3.3 | 33.22 ± 3.0 | <0.001 |
|  | Low-GNX | -54.88 ± 7.5 | -21.60 ± 6.9 | 2.04 ± 6.4 | 17.64 ± 6.9 | 28.97 ± 7.9 | 35.27 ± 7.0 | 40.32 ± 5.7 |  |
|  | Mid-GNX | -46.75 ± 5.3 | -30.79 ± 4.6 | -8.77 ± 5.6 | 10.69 ± 4.2 | 21.16 ± 4.7 | 26.13 ± 4.7 | 33.69 ± 5.8 |  |
|  | High-GNX | - 56.60 ± 5.5 | -38.22 ± 2.2* | -20.11 ± 4.1* | -6.28 ± 5.0* | 4.87 ± 2.9* | 11.94 ± 3.7* | 18.45 ± 3.4* |  |
| Female | - | -50.60 ± 9.9 | -18.73 ± 4.6 | 4.52 ± 5.0 | 15.32 ± 4.2 | 26.06 ± 6.4 | 34.04 ± 6.6 | 40.23 ± 5.9 | <0.001 |
|  | Low-GNX | -66.60 ± 7.1 | -31.47 ± 4.5* | -9.04 ± 3.4* | 5.67 ± 2.55 | 15.27 ± 3.6 | 24.63 ± 3.0 | 30.38 ± 3.9 |  |
|  | Mid-GNX | -57.62 ± 7.9 | -42.15 ± 9.1* | -11.92 ± 9.1 | 3.10 ± 8.1 | 12.92 ± 6.0 | 22.42 ± 5.0 | 29.67 ± 5.1 |  |
|  | High-GNX | -62.87 ± 6.3 | -57.08 ± 3.7* | -33.33 ± 4.8* | -6.95 ± 7.6* | 2.22 ± 7.4* | 8.63 ± 8.0* | 14.35 ± 8.8* |  |

ST2. Ponderal Index (adjusted means and SEM)

| Sex | Treatment | 0 | 4 | 7 | Model p-value |
| --- | --- | --- | --- | --- | --- |
| Male | - | 11.90 ± 0.46 | 10.90 ± 0.21 | 11.66 ± 0.26 | <0.001 |
|  | Low-GNX | 12.06 ± 0.26 | 10.58 ± 0.33 | 11.63 ± 0.34 |  |
|  | Mid-GNX | 11.77 ± 0.25 | 10.60 ± 0.27 | 11.50 ± 0.23 |  |
|  | High-GNX | 11.58 ± 0.41 | 10.02 ± 0.37* | 11.03 ± 0.23 |  |
| Female | - | 12.07 ± 0.31 | 10.73 ± 0.24 | 11.43 ± 0.35 | <0.001 |
|  | Low-GNX | 11.57 ± 0.18 | 10.56 ± 0.19 | 11.40 ± 0.25 |  |
|  | Mid-GNX | 11.66 ± 0.08 | 10.29 ± 0.27 | 11.42 ± 0.35 |  |
|  | High-GNX | 12.05 ± 0.38 | 10.90 ± 0.22 | 11.38 ± 0.20 |  |

ST3. Daily supplemental feeding (adjusted means and SEM)

| Sex | Treatment | 0 | 1 | 2 | 3 | 4 | 5 | 6 | Model p-value |
| --- | --- | --- | --- | --- | --- | --- | --- | --- | --- |
| Male | - | 3.82 ± 0.16 | 4.11 ± 0.20 | 3.01 ± 0.50 | 1.04 ± 0.31 | 0.99 ± 0.30 | 0.36 ± 0.17 | 0.20 ± 0.12 | <0.001 |
|  | Low-GNX | 3.72 ± 0.24 | 4.38 ± 0.17 | 2.42 ± 0.24 | 0.77 ± 0.34 | 0.27 ± 0.24 | 0.22 ± 0.20 | 0.00 |  |
|  | Mid-GNX | 3.98 ± 0.21 | 4.18 ± 0.14 | 3.87 ± 0.14 | 2.17 ± 0.38* | 0.63 ± 0.28 | 0.40 ± 0.37 | 0.00 |  |
|  | High-GNX | 4.15 ± 0.29 | 4.37 ± 0.16 | 3.78 ± 0.23 | 2.65 ± 0.49* | 1.40 ± 0.76 | 0.77 ± 0.57 | 0.10 ± 0.09 |  |
| Female | - | 3.90 ± 0.13 | 3.87 ± 0.20 | 2.80 ± 0.48 | 1.61 ± 0.37 | 0.89 ± 0.30 | 0.31 ± 0.16 | 0.17 ± 0.10 | <0.001 |
|  | Low-GNX | 4.02 ± 0.27 | 4.17 ± 0.20 | 3.73 ± 0.16 | 1.85 ± 0.39 | 0.60 ± 0.27 | 0.37 ± 0.24 | 0.00 |  |
|  | Mid-GNX | 3.87 ± 0.18 | 4.07 ± 0.16 | 3.67 ± 0.10 | 2.15 ± 0.36 | 1.78 ± 0.64 | 0.62 ± 0.48 | 0.00 |  |
|  | High-GNX | 4.16 ± 0.26 | 4.50 ± 0.13* | 5.26 ± 0.56* | 3.40 ± 0.99 | 1.96 ± 1.06 | 2.34 ± 1.48 | 2.28 ± 1.46 |  |

ST4. Wellbeing post-dose (adjusted means and SEM)

| Sex | Treatment | 0 | 1 | 2 | 3 | 4 | 5 | 6 | Model p-value |
| --- | --- | --- | --- | --- | --- | --- | --- | --- | --- |
| Male | - | 8.68 ± 0.18 | 9.54 ± 0.31 | 10.36 ± 0.34 | 11.32 ± 0.37 | 11.79 ± 0.20 | 12.00 ± 0 | 12.00 ± 0 | <0.001 |
|  | Low-GNX | 8.17 ± 0.42 | 8.63 ± 0.44 | 11.29 ± 0.41 | 11.63 ± 0.17 | 11.95 ± 0.06 | 11.95 ± 0.06 | 11.95 ± 0.06 |  |
|  | Mid-GNX | 7.88 ± 0.30* | 9.17 ± 0.36 | 10.63 ± 0.43 | 11.17 ± 0.37 | 11.67 ± 0.14 | 12.00 ± 0 | 12.00 ± 0 |  |
|  | High-GNX | 7.38 ± 0.35* | 8.21 ± 0.58* | 9.88 ± 0.57 | 10.88 ± 0.33 | 11.63 ± 0.16 | 12.00 ± 0 | 11.92 ± 0.08 |  |
| Female | - | 8.57 ± 0.33 | 9.75 ± 0.27 | 11.00 ± 0.27 | 11.75 ± 0.17 | 11.89 ± 0.07 | 12.00 ± 0 | 12.00 ± 0 | <0.001 |
|  | Low-GNX | 8.29 ± 0.34 | 8.83 ± 0.25* | 10.79 ± 0.34 | 11.33 ± 0.26 | 11.83 ± 0.15 | 11.83 ± 0.15 | 11.96 ± 0.04 |  |
|  | Mid-GNX | 8.04 ± 0.41 | 8.63 ± 0.19* | 9.92 ± 0.22* | 10.83 ± 0.33* | 11.75 ± 0.12 | 12.00 ± 0 | 12.00 ± 0 |  |
|  | High-GNX | 6.95 ± 0.60* | 6.90 ± 0.59* | 8.20 ± 0.47* | 10.60 ± 0.23* | 11.30 ± 0.16* | 11.70 ± 0.27 | 11.55 ± 0.40 |  |

ST5. Body and organ weights at term equivalence (adjusted means and SEM)

| Sex | Delivery | Treatment | Wgt 0 | Brain Wgt | Hippo. Wgt | Cereb. Wgt | Liver Wgt | Heart Wgt | Kidney Wgt | Adrenals Wgt | Sub. Cut. Fat Wgt | Visc. Fat Wgt |
| --- | --- | --- | --- | --- | --- | --- | --- | --- | --- | --- | --- | --- |
| Male | Term |  | 97.2 ± 4.7 | 2.61 ± 0.05 | 0.112 ± 0.005 | 0.141 ± 0.010 | 3.97 ± 0.25 | 0.395 ± 0.017 | 0.83 ± 0.051 | 0.034 ± 0.022 | 1.40 ± 0.10 | 0.837 ± 0.045 |
|  | Preterm | - | 89.78 ± 4.8 | 2.46 ± 0.05 | 0.115 ± 0.005 | 0.158 ± 0.011 | 2.84 ± 0.23 | 0.320 ± 0.018 | 0.94 ± 0.054 | 0.034 ± 0.024 | 0.83 ± 0.09* | 0.312 ± 0.042* |
|  |  | Low-GNX | 100.1 ± 5.0 | 2.43 ± 0.06 | 0.110 ± 0.005 | 0.150 ± 0.012 | 3.70 ± 0.23 | 0.336 ± 0.020 | 1.35 ± 0.059* | 0.044 ± 0.026* | 1.27 ± 0.09 | 0.385 ± 0.040* |
|  |  | Mid-GNX | 92.5 ± 4.9 | 2.47 ± 0.06 | 0.114 ± 0.005 | 0.151 ± 0.012 | 3.52 ± 0.23 | 0.330 ± 0.020 | 1.34 ± 0.057* | 0.048 ± 0.025* | 1.15 ± 0.09 | 0.307 ± 0.041* |
|  |  | High-GNX | 86.0 ± 5.0 | 2.43 ± 0.06 | 0.104 ± 0.005 | 0.148 ± 0.012 | 3.26 ± 0.23 | 0.322 ± 0.020 | 1.23 ± 0.058* | 0.046 ± 0.025* | 1.04 ± 0.09* | 0.323 ± 0.042* |
| Model p-value (Males) | | | 0.281 | 0.224 | 0.529 | 0.823 | 0.067 | 0.124 | 0.004 | 0.027 | 0.016 | 0.002 |
| Female | Term |  | 98.6 ± 5.2 | 2.52 ± 0.05 | 0.123 ± 0.006 | 0.158 ± 0.008 | 3.91 ± 0.28 | 0.386 ± 0.021 | 0.85 ± 0.071 | 0.037 ± 0.003 | 1.22 ± 0.10 | 0.663 ± 0.048 |
|  | Preterm | - | 88.4 ± 5.2 | 2.37 ± 0.05 | 0.097 ± 0.006 | 0.143 ± 0.008 | 3.17 ± 0.28 | 0.304 ± 0.021 | 0.98 ± 0.071 | 0.039 ± 0.003 | 0.80 ± 0.10 | 0.295 ± 0.042* |
|  |  | Low-GNX | 86.4 ± 5.6 | 2.38 ± 0.06 | 0.108 ± 0.007 | 0.163 ± 0.008 | 2.95 ± 0.30 | 0.300 ± 0.023 | 1.24 ± 0.077 | 0.049 ± 0.003 | 0.95 ± 0.10 | 0.269 ± 0.042* |
|  |  | Mid-GNX | 80.7 ± 5.6 | 2.35 ± 0.06 | 0.108 ± 0.007 | 0.158 ± 0.008 | 2.88 ± 0.33 | 0.268 ± 0.023 | 1.14 ± 0.077 | 0.047 ± 0.003 | 0.88 ± 0.10 | 0.283 ± 0.043* |
|  |  | High-GNX | 77.5 ± 6.1 | 2.25 ± 0.06 | 0.105 ± 0.007 | 0.136 ± 0.009 | 2.75 ± 0.37 | 0.273 ± 0.025 | 1.18 ± 0.084 | 0.049 ± 0.004 | 0.73 ± 0.11 | 0.200 ± 0.046* |
| Model p-value (Females) | | | 0.201 | 0.144 | 0.194 | 0.134 | 0.185 | 0.058 | 0.058 | 0.137 | 0.098 | 0.007 |

ST6. Body measurements at term equivalence (adjusted means and SEM)

| Sex | Delivery | Treatment | Nose-Rump | Head Length | Crown Rump | Hind Limb | Hock Toe | Head Circ | Neck Circ | Chest Circ | Abdo. Circ |
| --- | --- | --- | --- | --- | --- | --- | --- | --- | --- | --- | --- |
| Male | Term |  | 168.6 ± 2.7 | 53.1 ± 1.9 | 130.9 ± 2.4 | 38.0 ± 0.76 | 37.3 ± 0.5 | 87.8 ± 1.8 | 74.9 ± 2.0 | 92.6 ± 2.6 | 96.6 ± 3.4 |
|  | Preterm | - | 158.1 ± 2.9 | 48.4 ± 1.9 | 126.4 ± 2.5 | 35.9 ± 0.7 | 34.6 ± 0.5* | 88.7 ± 2.0 | 69.4 ± 1.9 | 88.6 ± 2.5 | 93.8 ± 3.3 |
|  |  | Low-GNX | 164.5 ± 3.2 | 50.8 ± 1.9 | 132.1 ± 2.6 | 36.3 ± 0.7 | 35.0 ± 0.5* | 86.4 ± 2.1 | 72.6 ± 1.9 | 92.4 ± 2.5 | 96.8 ± 3.2 |
|  |  | Mid-GNX | 163.4 ± 3.1 | 49.5 ± 1.9 | 129.3 ± 2.6 | 36.3 ± 0.7 | 35.0 ± 0.5* | 86.4 ± 2.0 | 70.0 ± 2.0   \|  \| \| --- \| | 93.3 ± 2.6 | 98.5 ± 3.3 |
|  |  | High-GNX | 157.7 ± 3.1 | 48.4 ± 1.9 | 126.3 ± 2.6 | 35.3 ± 0.7 | 34.3 ± 0.5* | 85.3 ± 2.0 | 67.5 ± 2.0 | 90.7 ± 2.6 | 95.7 ± 3.3 |
| Model p-value (Males) | | | 0.141 | 0.404 | 0.369 | 0.185 | 0.036 | 0.782 | 0.167 | 0.568 | 0.784 |
| Female | Term |  | 168.4 ± 2.9 | 53.4 ± 1.6 | 131.6 ± 2.8 | 38.3 ± 0.7 | 37.3 ± 0.6 | 88.9 ± 1.3 | 74.7 ± 2.7 | 90.4 ± 2.5 | 94.9 ± 3.4 |
|  | Preterm | - | 158.9 ± 2.9 | 47.8 ± 1.6 | 126.9 ± 2.8 | 36.0 ± 0.7 | 34.6 ± 0.6 | 85.3 ± 1.3 | 69.1 ± 2.5 | 89.5 ± 2.5 | 93.8 ± 3.4 |
|  |  | Low-GNX | 159.5 ± 3.2 | 47.1 ± 1.6 | 125.8 ± 3.1 | 35.9 ± 0.8 | 34.3 ± 0.7 | 83.8 ± 1.4 | 66.2 ± 2.6 | 88.9 ± 2.6 | 93.2 ± 3.6 |
|  |  | Mid-GNX | 157.5 ± 3.2 | 51.7 ± 1.6 | 122.5 ± 3.1 | 35.4 ± 0.8 | 34.2 ± 0.7 | 82.0 ± 1.4 | 69.3 ± 2.7 | 85.8 ± 2.6 | 90.6 ± 3.6 |
|  |  | High-GNX | 154.6 ± 3.5 | 46.2 ± 1.8 | 119.8 ± 3.4 | 34.6 ± 0.9 | 33.7 ± 0.7 | 81.8 ± 1.5 | 64.3 ± 2.9 | 86.5 ± 2.9 | 90.8 ± 3.9 |
| Model p-value (Females) | | | 0.139 | 0.081 | 0.212 | 0.115 | 0.060 | 0.057 | 0.226 | 0.693 | 0.889 |

ST7. Organ weights and body measurements at term equivalence for vehicle-treated preterm versus untreated preterm control neonates (raw means and SEM).

|  | Male | | | Female | | |
| --- | --- | --- | --- | --- | --- | --- |
|  | Preterm (CON) | Preterm (VEH) | p-value | Preterm (CON) | Preterm (VEH) | p-value |
| Wgt 0 | 90.8 ± 7.2 | 91.2 ± 2.1 | 0.96 | 88.4 ± 5.8 | 82.3 ± 4.6 | 0.43 |
| Brain Wgt | 2.46 ± 0.08 | 2.45 ± 0.04 | 0.87 | 2.37 ± 0.04 | 2.33 ± 0.04 | 0.60 |
| Hippo. Wgt | 0.116 ± 0.004 | 0.109 ± 0.006 | 0.27 | 0.097 ± 0.004 | 0.106 ± 0.009 | 0.36 |
| Cereb. Wgt | 0.158 ± 0.015 | 0.164 ± 0.007 | 0.75 | 0.142 ± 0.007 | 0.161 ± 0.005 | 0.063 |
| Liver Wgt | 2.92 ± 0.24 | 3.14 ± 0.16 | 0.48 | 3.17 ± 0.34 | 2.98 ± 0.37 | 0.70 |
| Heart Wgt | 0.320 ± 0.023 | 0.320 ± 0.010 | 0.99 | 0.304 ± 0.027 | 0.288 ± 0.026 | 0.67 |
| Kidney Wgt | 0.94 ± 0.073 | 1.27 ± 0.025* | 0.0023 | 0.98 ± 0.081 | 1.25 ± 0.065* | 0.032 |
| Adrenals Wgt | 0.034 ± 0.002 | 0.044 ± 0.002* | 0.012 | 0.039 ± 0.002 | 0.046 ± 0.003 | 0.12 |
| Sub. Cut. Fat Wgt | 0.90 ± 0.13 | 1.16 ± 0.12 | 0.19 | 0.79 ± 0.12 | 0.83 ± 0.10 | 0.77 |
| Visc. Fat Wgt | 0.323 ± 0.060 | 0.295 ± 0.047 | 0.73 | 0.288 ± 0.056 | 0.214 ± 0.052 | 0.41 |
| Nose-Rump | 158.6 ± 3.4 | 163.2 ± 1.9 | 0.28 | 158.9 ± 2.8 | 158.3 ± 2.6 | 0.89 |
| Head Length | 48.6 ± 2.3 | 50.5 ± 2.1 | 0.56 | 47.6 ± 1.2 | 48.2 ± 1.8 | 0.78 |
| Crown Rump | 127.0 ± 3.2 | 127.8 ± 1.9 | 0.83 | 126.9 ± 3.0 | 126.0 ± 2.7 | 0.84 |
| Hind Limb | 35.9 ± 0.8 | 35.3 ± 0.6 | 0.61 | 36.0 ± 0.7 | 35.3 ± 0.5 | 0.48 |
| Hock Toe | 34.6 ± 0.6 | 34.5 ± 0.2 | 0.92 | 34.6 ± 0.6 | 34.0 ± 0.6 | 0.53 |
| Head Circ. | 88.8 ± 2.9 | 86.2 ± 1.2 | 0.42 | 85.3 ± 1.1 | 82.8 ± 1.1 | 0.15 |
| Neck Circ. | 70.3 ± 2.5 | 68.3 ± 1.4 | 0.53 | 68.3 ± 4.3 | 65.2 ± 2.9 | 0.57 |
| Chest Circ. | 90.0 ± 3.4 | 90.5 ± 2.0 | 0.91 | 89.3 ± 2.3 | 87.0 ± 2.4 | 0.51 |
| Abdo Circ. | 94.9 ± 4.2 | 98.2 ± 4.1 | 0.59 | 93.6 ± 3.5 | 88.5 ± 2.8 | 0.29 |

ST8. Immunohistochemistry (adjusted means and SEM)

| Sex | Delivery | Treatment | MBP (CA1) | MBP (SCWM) | NeuN (CA1) | NeuN (SCWM) |
| --- | --- | --- | --- | --- | --- | --- |
| Male | Term |  | 13.06 ± 0.51 | 34.27 ± 0.84 | 16.62 ± 0.44 | 21.93 ± 0.78 |
|  | Preterm | - | 11.68 ± 0.55 | 32.97 ± 0.90 | 15.97 ± 0.51 | 22.10 ± 0.90 |
|  |  | Low-GNX | 10.76 ± 0.59 | 32.12 ± 0.97 | 15.46 ± 0.51 | 21.71 ± 0.90 |
|  |  | Mid-GNX | 11.05 ± 0.59 | 30.84 ± 0.97 | 16.54 ± 0.51 | 21.85 ± 0.90 |
|  |  | High-GNX | 12.06 ± 0.59 | 33.08 ± 0.97 | 15.82 ± 0.51 | 22.08 ± 0.90 |
| Model p-value (Males) | | | 0.146 | 0.243 | 0.478 | 0.997 |
| Female | Term |  | 12.98 ± 0.68 | 34.73 ± 1.55 | 16.76 ± 0.56 | 22.02 ± 0.64 |
|  | Preterm | - | 11.02 ± 0.68 | 30.41 ± 1.41 | 16.54 ± 0.56 | 21.75 ± 0.64 |
|  |  | Low-GNX | 10.99 ± 0.80 | 31.14 ± 1.64 | 15.28 ± 0.66 | 21.12 ± 0.75 |
|  |  | Mid-GNX | 11.03 ± 0.73 | 29.94 ± 1.51 | 16.26 ± 0.66 | 21.70 ± 0.75 |
|  |  | High-GNX | 10.25 ± 0.80 | 29.00 ± 1.65 | 15.67 ± 0.66 | 22.73 ± 0.75 |
| Model p-value (Females) | | | 0.223 | 0.246 | 0.494 | 0.674 |

ST9. mRNA Relative Expressions (adjusted means and SEM)

|  | Males | | | | | | Females | | | | | |
| --- | --- | --- | --- | --- | --- | --- | --- | --- | --- | --- | --- | --- |
| Gene | Term | Preterm | Preterm  Low-GNX | Preterm  Mid-GNX | Preterm  High-GNX | Model p-value | Term | Preterm | Preterm  Low-GNX | Preterm  Mid-GNX | Preterm  High-GNX | Model p-value |
| *ABAT* | 0.981 ± 0.037 | 0.971 ± 0.039 | 1.019 ± 0.046 | 0.988 ± 0.042 | 0.926 ± 0.042 | 0.685 | 0.987 ± 0.047 | 0.933 ± 0.050 | 1.049 ± 0.050 | 0.941 ± 0.050 | 0.908 ± 0.055 | 0.421 |
| *CALB1* | 0.892 ± 0.060 | 1.100 ± 0.065 | 1.259 ± 0.077 | 1.037 ± 0.070 | 1.074 ± 0.070 | 0.115 | 0.933 ± 0.093 | 1.131 ± 0.099 | 1.168 ± 0.107 | 1.052 ± 0.099 | 1.147 ± 0.108 | 0.510 |
| *CSPG4* | 0.727 ± 0.106 | 0.300 ± 0.074 | 0.270 ± 0.073 | 0.142 ± 0.074* | 0.176 ± 0.074* | 0.033 | 0.630 ± 0.059 | 0.158 ± 0.063* | 0.131 ± 0.063* | 0.139 ± 0.063* | 0.181 ± 0.078* | 0.015 |
| *DLG4* | 0.989 ± 0.039 | 0.730 ± 0.036* | 0.722 ± 0.036* | 0.612 ± 0.035* | 0.610 ± 0.035* | 0.011 | 0.934 ± 0.046 | 0.713 ± 0.050 | 0.710 ± 0.049 | 0.686 ± 0.050 | 0.669 ± 0.054 | 0.055 |
| *GABRA1* | 1.098 ± 0.068 | 1.026 ± 0.073 | 1.275 ± 0.088 | 1.150 ± 0.078 | 1.095 ± 0.078 | 0.394 | 1.054 ± 0.053 | 1.016 ± 0.057 | 0.913 ± 0.057 | 1.065 ± 0.057 | 1.076 ± 0.062 | 0.370 |
| *GABRA2* | 1.059 ± 0.058 | 1.070 ± 0.061 | 1.154 ± 0.073 | 1.055 ± 0.066 | 1.268 ± 0.066 | 0.263 | 0.940 ± 0.046 | 1.141 ± 0.047 | 1.074 ± 0.046 | 1.160 ± 0.046 | 1.043 ± 0.050 | 0.092 |
| *GABRA3* | 0.844 ± 0.109 | 0.956 ± 0.115 | 1.271 ± 0.137 | 0.973 ± 0.122 | 0.990 ± 0.122 | 0.334 | 0.907 ± 0.125 | 1.007 ± 0.133 | 1.033 ± 0.131 | 1.007 ± 0.132 | 0.928 ± 0.144 | 0.938 |
| *GABRA4* | 1.089 ± 0.065 | 1.108 ± 0.067 | 1.426 ± 0.075* | 1.293 ± 0.068 | 1.006 ± 0.069 | 0.036 | 1.108 ± 0.066 | 1.089 ± 0.071 | 1.117 ± 0.070 | 1.191 ± 0.070 | 1.051 ± 0.077 | 0.708 |
| *GABRA5* | 1.070 ± 0.042 | 1.051 ± 0.045 | 1.137 ± 0.053 | 1.123 ± 0.048 | 1.161 ± 0.048 | 0.505 | 0.975 ± 0.048 | 1.131 ± 0.052 | 1.241 ± 0.052 | 1.178 ± 0.052 | 1.200 ± 0.057 | 0.070 |
| *GABRD* | 0.907 ± 0.041 | 0.859 ± 0.044 | 0.863 ± 0.052 | 0.798 ± 0.048 | 0.732 ± 0.048 | 0.230 | 0.864 ± 0.046 | 0.765 ± 0.049 | 0.786 ± 0.049 | 0.813 ± 0.049 | 0.704 ± 0.054 | 0.353 |
| *GABRG2* | 0.984 ± 0.064 | 1.182 ± 0.068 | 1.313 ± 0.081 | 1.145 ± 0.074 | 1.314 ± 0.074 | 0.107 | 1.106 ± 0.055 | 1.240 ± 0.059 | 1.127 ± 0.059 | 1.098 ± 0.059 | 1.254 ± 0.065 | 0.276 |
| *GAD1(67)* | 0.952 ± 0.044 | 0.912 ± 0.047 | 0.950 ± 0.056 | 0.910 ± 0.051 | 0.844 ± 0.051 | 0.598 | 0.872 ± 0.048 | 0.849 ± 0.051 | 0.928 ± 0.051 | 0.907 ± 0.051 | 0.746 ± 0.063 | 0.356 |
| *GAD2* | 0.928 ± 0.028 | 0.955 ± 0.029 | 0.976 ± 0.032 | 0.881 ± 0.029 | 0.868 ± 0.029 | 0.184 | 0.874 ± 0.027 | 0.841 ± 0.030 | 0.914 ± 0.030 | 0.929 ± 0.033 | 0.745 ± 0.033* | 0.049 |
| *GLS1* | 0.940 ± 0.040 | 0.930 ± 0.046 | 0.983 ± 0.050 | 0.965 ± 0.046 | 0.999 ± 0.046 | 0.799 | 0.915 ± 0.051 | 1.034 ± 0.055 | 1.054 ± 0.055 | 1.020 ± 0.055 | 1.016 ± 0.060 | 0.459 |
| *GRIN1* | 0.741 ± 0.055 | 0.469 ± 0.057* | 0.431 ± 0.063* | 0.366 ± 0.058* | 0.407 ± 0.058* | 0.05 | 0.528 ± 0.034 | 0.413 ± 0.036 | 0.414 ± 0.036 | 0.434 ± 0.036 | 0.411 ± 0.039 | 0.231 |
| *GRIN2C* | 1.217 ± 0.061 | 0.707 ± 0.055* | 0.623 ± 0.052* | 0.522 ± 0.053* | 0.454 ± 0.053* | 0.005 | 1.103 ± 0.055 | 0.552 ± 0.053* | 0.558 ± 0.051* | 0.574 ± 0.052* | 0.499 ± 0.057* | 0.003 |
| *INA* | 0.897 ± 0.037 | 0.618 ± 0.040* | 0.607 ± 0.047* | 0.524 ± 0.043* | 0.549 ± 0.043* | 0.011 | 0.797 ± 0.051 | 0.608 ± 0.055 | 0.548 ± 0.055 | 0.575 ± 0.055 | 0.488 ± 0.060 | 0.056 |
| *MBP* | 0.846 ± 0.052 | 0.597 ± 0.054* | 0.547 ± 0.059* | 0.504 ± 0.054* | 0.495 ± 0.054* | 0.050 | 0.739 ± 0.048 | 0.496 ± 0.052* | 0.432 ± 0.052* | 0.480 ± 0.052* | 0.465 ± 0.057* | 0.035 |
| *OLIG2* | 0.776 ± 0.089 | 0.473 ± 0.095 | 0.517 ± 0.113 | 0.451 ± 0.103 | 0.411 ± 0.103 | 0.201 | 0.634 ± 0.067 | 0.445 ± 0.055 | 0.391 ± 0.053 | 0.445 ± 0.054 | 0.355 ± 0.057 | 0.123 |
| *PVALB* | 1.081 ± 0.106 | 0.652 ± 0.114 | 0.650 ± 0.135 | 0.810 ± 0.123 | 0.664 ± 0.123 | 0.173 | 1.008 ± 0.063 | 0.563 ± 0.058* | 0.476 ± 0.055* | 0.615 ± 0.056* | 0.500 ± 0.061* | 0.011 |
| *RBFOX3* | 0.912 ± 0.085 | 0.570 ± 0.077 | 0.652 ± 0.074 | 0.547 ± 0.074 | 0.459 ± 0.075 | 0.068 | 0.641 ± 0.033 | 0.524 ± 0.036 | 0.566 ± 0.036 | 0.570 ± 0.036 | 0.490 ± 0.039 | 0.164 |
| *SLC1A2* | 1.194 ± 0.069 | 0.912 ± 0.073 | 0.879 ± 0.087 | 0.897 ± 0.079 | 0.989 ± 0.079 | 0.021 | 1.301 ± 0.083 | 0.812 ± 0.090* | 0.790 ± 0.090* | 0.839 ± 0.090* | 0.796 ± 0.098* | 0.060 |
| *SLC32A1* | 0.471 ± 0.080 | 0.095 ± 0.056* | 0.198 ± 0.055* | 0.153 ± 0.055* | 0.173 ± 0.055* | 0.006 | 0.224 ± 0.028 | 0.141 ± 0.023 | 0.110 ± 0.022 | 0.126 ± 0.022 | 0.093 ± 0.023 | 0.086 |
| *SST* | 0.701 ± 0.094 | 0.352 ± 0.068 | 0.344 ± 0.065 | 0.275 ± 0.066 | 0.297 ± 0.066 | 0.108 | 0.465 ± 0.031 | 0.279 ± 0.034* | 0.237 ± 0.034* | 0.282 ± 0.034* | 0.289 ± 0.037* | 0.022 |
| *VEGFA* | 0.979 ± 0.066 | 0.542 ± 0.070* | 0.392 ± 0.083* | 0.321 ± 0.076* | 0.326 ± 0.076* | 0.010 | 0.854 ± 0.047 | 0.408 ± 0.051* | 0.427 ± 0.051* | 0.340 ± 0.051* | 0.245 ± 0.056* | 0.002 |
